# Fluctuation-Driven Neural Dynamics Reproduce *Drosophila* Locomotor Patterns

**DOI:** 10.1371/journal.pcbi.1004577

**Published:** 2015-11-23

**Authors:** Andrea Maesani, Pavan Ramdya, Steeve Cruchet, Kyle Gustafson, Richard Benton, Dario Floreano

**Affiliations:** 1 Institute of Microengineering, École Polytechnique Fédérale de Lausanne, Lausanne, Switzerland; 2 Center for Integrative Genomics, Faculty of Biology and Medicine, University of Lausanne, Lausanne, Switzerland; 3 The Institute of Bioengineering, School of Life Sciences, École Polytechnique Fédérale de Lausanne, Lausanne, Switzerland; Imperial College London, UNITED KINGDOM

## Abstract

The neural mechanisms determining the timing of even simple actions, such as when to walk or rest, are largely mysterious. One intriguing, but untested, hypothesis posits a role for ongoing activity fluctuations in neurons of central action selection circuits that drive animal behavior from moment to moment. To examine how fluctuating activity can contribute to action timing, we paired high-resolution measurements of freely walking *Drosophila melanogaster* with data-driven neural network modeling and dynamical systems analysis. We generated fluctuation-driven network models whose outputs—locomotor bouts—matched those measured from sensory-deprived *Drosophila*. From these models, we identified those that could also reproduce a second, unrelated dataset: the complex time-course of odor-evoked walking for genetically diverse *Drosophila* strains. Dynamical models that best reproduced both *Drosophila* basal and odor-evoked locomotor patterns exhibited specific characteristics. First, ongoing fluctuations were required. In a stochastic resonance-like manner, these fluctuations allowed neural activity to escape stable equilibria and to exceed a threshold for locomotion. Second, odor-induced shifts of equilibria in these models caused a depression in locomotor frequency following olfactory stimulation. Our models predict that activity fluctuations in action selection circuits cause behavioral output to more closely match sensory drive and may therefore enhance navigation in complex sensory environments. Together these data reveal how simple neural dynamics, when coupled with activity fluctuations, can give rise to complex patterns of animal behavior.

## Author Summary

The brain is never quiet. Even in the absence of environmental cues, neurons receive and produce an ongoing barrage of fluctuating signals. These fluctuations are well studied in the sensory periphery but their potential influence on central circuits and behavior are unknown. In particular, activity fluctuations in action selection circuits—neural populations that drive an animal’s actions from moment to moment—may strongly influence behavior. To shed light on the influence of activity fluctuations on action timing, we developed a computational approach for automatically generating neural network models that reproduce large-scale, high-resolution behavioral measurements of freely walking *Drosophila melanogaster*. We found that models require stochastic activity fluctuations to reproduce complex *Drosophila* locomotor patterns. Specific fluctuation-driven dynamics allow these models to produce short and long bouts of locomotion in the absence of sensory cues and to reduce locomotor activity after sensory stimulation. These results support a role for ongoing activity fluctuations in the timing of animal behavior and reveal how behavioral shifts can be brought about through changes in the dynamics of neural circuits. Thus, simple dynamical mechanisms may underlie complex patterns of animal behavior.

## Introduction

Even in the absence of environmental cues, neurons receive and produce a barrage of fluctuating, ongoing signals. These fluctuations are both deterministic, reflecting a neuron’s embedding within complex dynamical networks, and random, arising from stochastic noise sources at synapses and ion channels [1,2]. Although the influence of these fluctuations on peripheral sensory processing is well studied [3–6], very little is known about how they may affect central circuits [7].

Action selection (AS) circuits [8], including ‘command’ neurons that drive behavior from moment to moment [9–11], may be particularly susceptible to activity fluctuations: they represent information bottlenecks where a relatively small number of neurons can have a disproportionately large influence on actions. The sensitivity of AS circuits to internally generated fluctuations in neural activity is suggested by ecological studies showing how intermittent patterns of walking and resting in animals [12] are well characterized by random walk models [13]. Similarly, behavioral transitions in *C*. *elegans* can be effectively captured using a tunable stochastic term within a deterministic mathematical framework [14].

While progress is being made [11,15,16], *in vivo* investigation of the dynamics of complex AS networks remains challenging. In this light, computational modeling can serve as an excellent starting point for generating theoretical predictions that guide *in vivo* studies. In particular, tools that exploit the power of neural network optimization and dynamical systems analysis [17] are gaining attention [18,19] for their ability to elucidate animal behavior [20,21] and the activity of neural ensembles [22,23].

In this study we used neural network optimization to infer the dynamics of AS circuits driving the locomotor walking patterns of *Drosophila melanogaster*. *Drosophila* is an attractive model organism for this type of investigation since its behaviors are increasingly well-described [24,25]. Previous studies of *Drosophila* locomotor patterning have predominantly focused on walking because this behavior has reproducible statistics and can be measured at high-throughput [26–29]. Importantly, due to their relatively small number of neurons as well as the availability of powerful genetic tools, *Drosophila* AS circuits are under intense investigation [11,16,30,31]. This raises the possibility of testing and further constraining computationally derived theoretical predictions.

Several models may explain how fluctuations in AS circuits influence neural activity and behavior. In the simplest, membrane potential fluctuations in AS neurons directly impact the firing of these neurons. Consequently, exceptionally high intensity fluctuations might cause command neurons to fire and initiate actions more frequently. However, this simple feed-forward framework ignores the highly interconnected nature of neural circuits within the central brain. Therefore, more complex dynamical models incorporating feedback may be more appropriate. However, the dynamical features that make central circuits more or less susceptible to the influence of activity fluctuations are unknown. These may include the location and number of stable and unstable equilibrium points in neural activity phase space.

To address this question we developed a method for automatically generating neural network models that reproduce measured animal behaviors. Our modeling approach relies on Continuous-Time Recurrent Neural Networks (CTRNNs): dynamical systems that share important properties with biological neural circuits [19,32]. These models are consequently more informative of *in vivo* circuit dynamics than other simple models like non-neuronal Markov [33,34] and random walk schemes [12,13]. We emphasize that the resulting neural networks are not intended to map directly onto the anatomy of *Drosophila* AS circuits. Instead they reveal emergent dynamics that represent theoretical predictions about *in vivo* circuit function.

To generate a behavioral dataset for constraining our models we first measured *Drosophila* basal (i.e., sensory deprived) and odor-evoked locomotor patterns. Next, to explore how neural activity fluctuations might be used to drive basal locomotion in sensory deprived *Drosophila*, we generated populations of neural network models whose virtual locomotor outputs reproduce the basal locomotor statistics of sensory deprived *Drosophila*. We next identified which of these models could also match, without changing their underlying dynamics, the odor-evoked locomotor patterns of genetically distinct *Drosophila* strains. Using dynamical systems analysis, we discovered that models that best reproduce *Drosophila* basal and odor-evoked locomotor patterns (i) require neural activity fluctuations and (ii) exhibit feedback-driven multistable dynamics that reorganize in response to sensory stimulation.

## Results

### Measuring *Drosophila* basal and odor-evoked locomotor patterns

**Fig 1:**
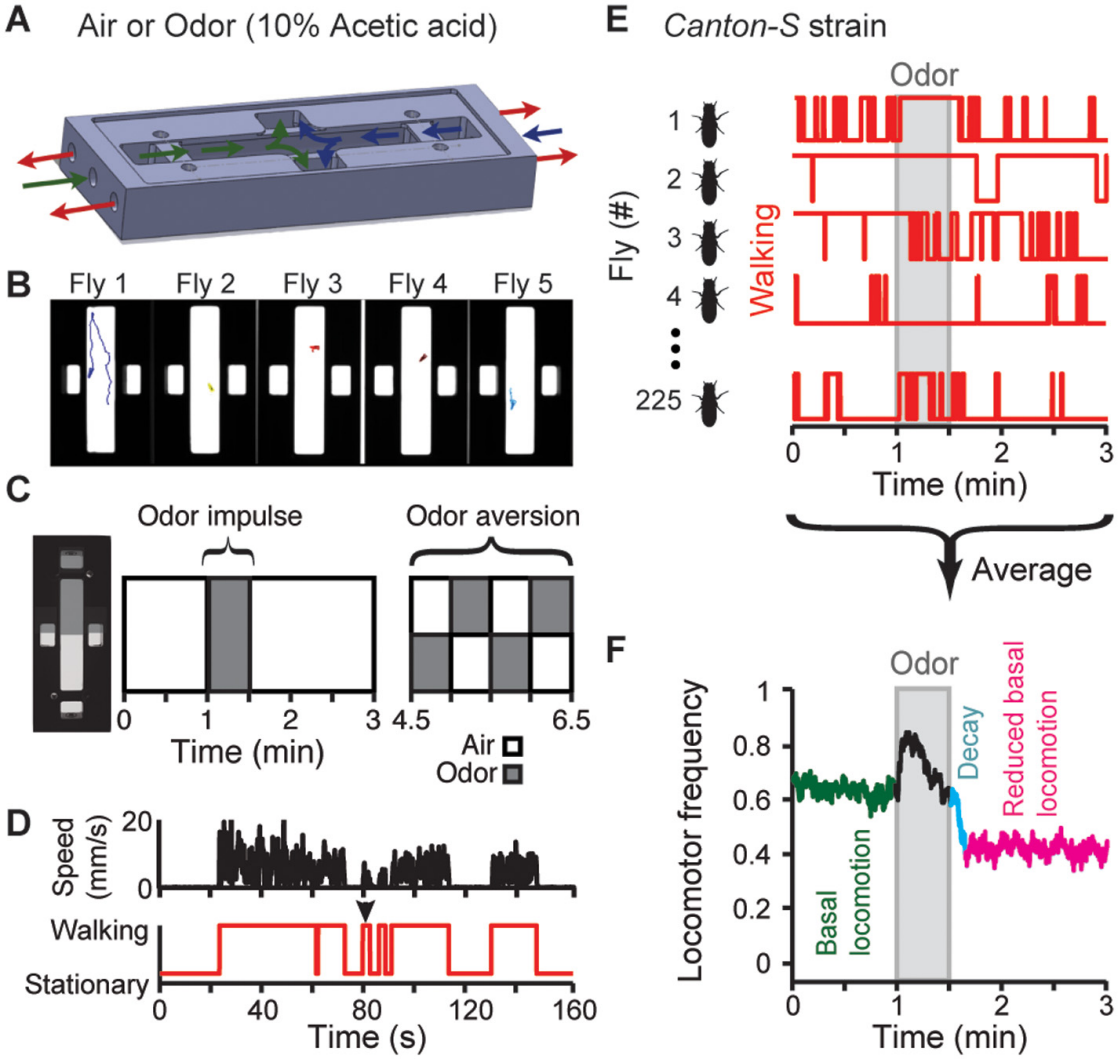
A high-resolution, high-throughput assay for measuring *Drosophila* locomotor patterns. (**A**) Schematic of planar behavioral arenas. Laminar flow of air or odor (10% acetic acid) is presented to either or both halves of the arena. Colored arrows indicate flow inlets and outlets (green/blue and red, respectively). (**B**) Camera-view of five experimental arenas and behavior tracking. Each fly is represented as a colored triangle. A colored line represents a fly’s location during the previous 10 s. (**C**) Schematic time-course of the behavioral experiment. We studied basal and odor-evoked locomotion as well as odor aversion for each fly. While not shown, during minutes 3–4.5, air flowed throughout the arena. (**D**) Speed time-series (black) were transformed into binary ‘Walking’ or ‘Stationary’ time-series (red). (**E**) Representative locomotor traces for five *Canton-S* strain flies during the odor impulse experiment. Flies were exposed to 60 s of air flow, 30 s of odor throughout the arena, and then 90 s of air flow. Behavior for each fly is shown in red. High values indicate walking while low values indicate stationary periods. (**F**) Locomotor traces averaged across 225 *Canton-S* flies during the odor impulse experiment. Prior to odor stimulation (grey bar) there is basal locomotion (green) followed by decay in locomotor frequency (cyan) to a reduced level of basal locomotion (magenta).

Our modeling approach relies on optimizing neural network parameters to match *Drosophila* data. Therefore, we quantified *Drosophila* locomotion with high temporal resolution by developing a high-throughput system combining synchronized video-capture at 20 frames per second (fps) [35,36], computer-controlled odor delivery (Fig 1A), and behavioral tracking [37] (Fig 1B) of the position and orientation of individual flies within a planar arena. Using this system we could study basal locomotion in the absence of visual [24,25], olfactory [38], gustatory [39,40], and time-varying mechanosensory/auditory [41] stimuli. In addition, to capture *Drosophila* behaviors driven by sensory cues [24,25], we used a system of valves to deliver precisely timed and spatially homogeneous odor stimuli (10% acetic acid [42]). Using these tools, we could acquire enough behavioral data to detect patterns in the highly variable behaviors of individual animals [43].

We performed two experiments for each individual fly (Fig 1C). In the first ‘odor impulse’ experiment we tracked 60 s of basal locomotion in the absence of any sensory stimulus, followed by 30 s of locomotor responses to uniform odor exposure, and finally 90 s of post-odor basal locomotion. In the second ‘odor aversion’ experiment we tracked locomotion for 2 min while presenting the aversive odorant on alternative sides of the arena in four separate 30 s periods. We performed experiments under dim far-red illumination, a wavelength of light for which flies are insensitive [44], to minimize the influence of visual cues on behavior.

While much information can be extracted from our measurements, we focused on the presence or absence of walking bouts since these most directly reflect the activity of AS circuits rather than downstream central pattern generators that control leg coordination and walking speed [45,46]. As in previous studies [47,48], we classified locomotor behaviors as intermittent walking or stationary intervals (Fig 1D) [12] by applying a cutoff to walking speed data (S1A and S1B Fig). As expected, basal locomotor behaviors for individual flies were unpredictable (Fig 1E) and characterized by bursts of locomotor activity separated by longer intervening periods of inactivity [26,28]. Therefore, to reveal patterns behind these highly variable behaviors, we averaged walking/stationary time-series across 225 genetically identical flies of a single, *Canton-S* strain. Prior to odor stimulation, flies exhibited a high basal locomotor frequency: many animals walked in the absence of salient sensory cues (Fig 1F, green, ‘Basal locomotion’). Upon odor presentation, locomotor frequency increased rapidly (Fig 1F, black). When the odor was removed (S1C Fig), locomotor frequency decayed (Fig 1F, cyan, ‘Decay’). Surprisingly, basal locomotor frequency did not simply return to the pre-odor rate but continued decaying to a substantially lower level (Fig 1F, magenta, ‘Reduced basal locomotion’).

### Measuring odor-evoked locomotor patterns across 98 *Drosophila* strains

**Fig 2:**
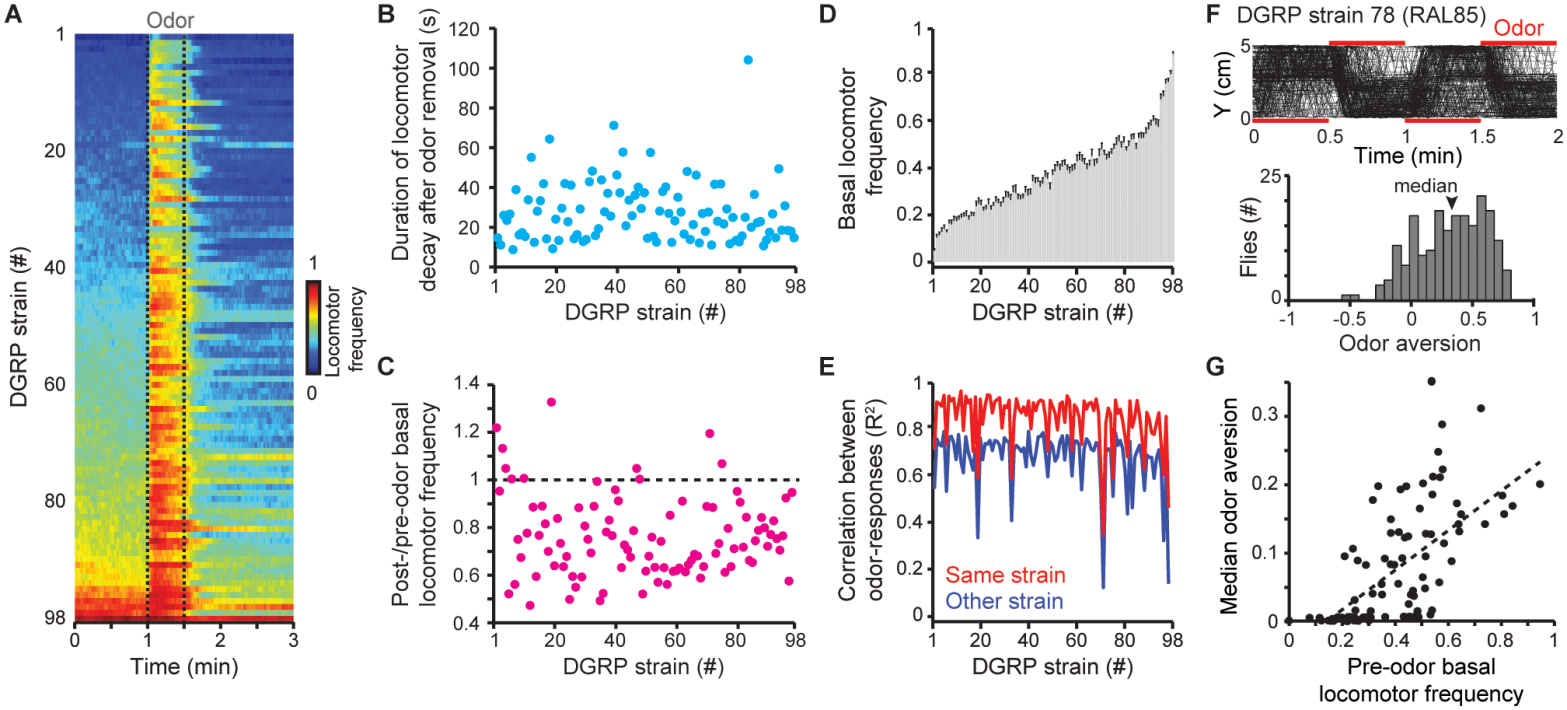
Locomotor patterns for genetically distinct *Drosophila* strains. (**A**) Locomotor frequency for 98 strains from the *Drosophila melanogaster* Genetic Reference Panel (DGRP) during the odor impulse experiment. Strains are sorted by average pre-odor basal locomotor frequency. (**B**) The duration of locomotor decay following odor removal for all DGRP strains. Strains are ordered as in panel **A**. (**C**) The ratio of post-odor to pre-odor basal locomotion for all DGRP strains. Strains are ordered as in panel **A**. The black dashed line indicates no change in basal locomotion. Values below this line represent reduced post-odor basal locomotor frequency. (**D**) The basal locomotor frequency for 65 randomly sampled flies (50% of flies for the strain with the smallest sample size: 130 flies) from each strain. The mean (light gray boxes) and standard deviation (black error bars) of 100 random samplings for each strain are shown. Strains are ordered as in panel **A**. (**E**) The correlation (R^2^) between odor-evoked locomotion time-series for groups of 65 randomly sampled flies taken from either the same strain (red) or from different *Drosophila* strains (blue). Strains are ordered as in panel **A**. The mean of 100 correlation measurements is shown. (**F**) Walking trajectories (black lines) along the long axis of the arena during an odor aversion experiment for 201 flies of the DGRP strain 78 (RAL85). Red bars indicate the half of the arena filled with odor. A histogram of odor aversion values for these flies is shown below. For each fly, odor aversion was calculated as the time spent in the odor zone subtracted from the time spent in air zone, divided by the total time of the odor aversion experiment. The median for this population of flies is indicated (black arrowhead). (**G**) A scatter plot showing the correlation between mean pre-odor basal locomotion and median odor aversion across all 98 strains (Pearson’s correlation coefficient R = 0.65, *P* < 10^−4^). A black dashed line indicates the best linear fit.

To examine the variation in these complex odor-evoked locomotor patterns, we tracked approximately 200 individuals from each of 98 genetically-distinct, inbred fly strains from the *Drosophila melanogaster* Genetic Reference Panel (DGRP) [49]. These experiments resulted in a behavioral dataset comprising 20,223 animals. Indeed, as for *Canton-S* flies (Fig 1F), across most of the DGRP strains (Fig 2A, see S1 Table for ‘RAL’ (Raleigh) strain IDs) we observed (i) basal locomotion (Fig 2D), (ii) post-odor decay of locomotion for periods ranging over an order of magnitude (Fig 2A and 2B), and (iii) in most strains, reductions in post-odor basal locomotion (Fig 2A and 2C, post-/pre-odor frequency < 1).

This rich behavioral diversity might reflect random experimental variation or, alternatively, intrinsic biological differences between each strain. To distinguish between these possibilities, we examined the reproducibility of locomotor patterns and found that average basal locomotion was highly consistent for each strain (Fig 2D). Similarly, the time-course of odor-evoked and post-odor locomotion more closely matched between flies of the same strain (Fig 2E, red) than between flies of different strains (Fig 2E, blue). Importantly, these simple locomotor characteristics are linked to more complex, ethologically relevant behaviors: median odor aversion (Fig 2F) was significantly correlated with basal locomotor frequency across all strains (Fig 2G, Pearson’s correlation coefficient R = 0.65, *P* < 10^−4^).

### Data-driven generation of dynamical neural network models

**Fig 3:**
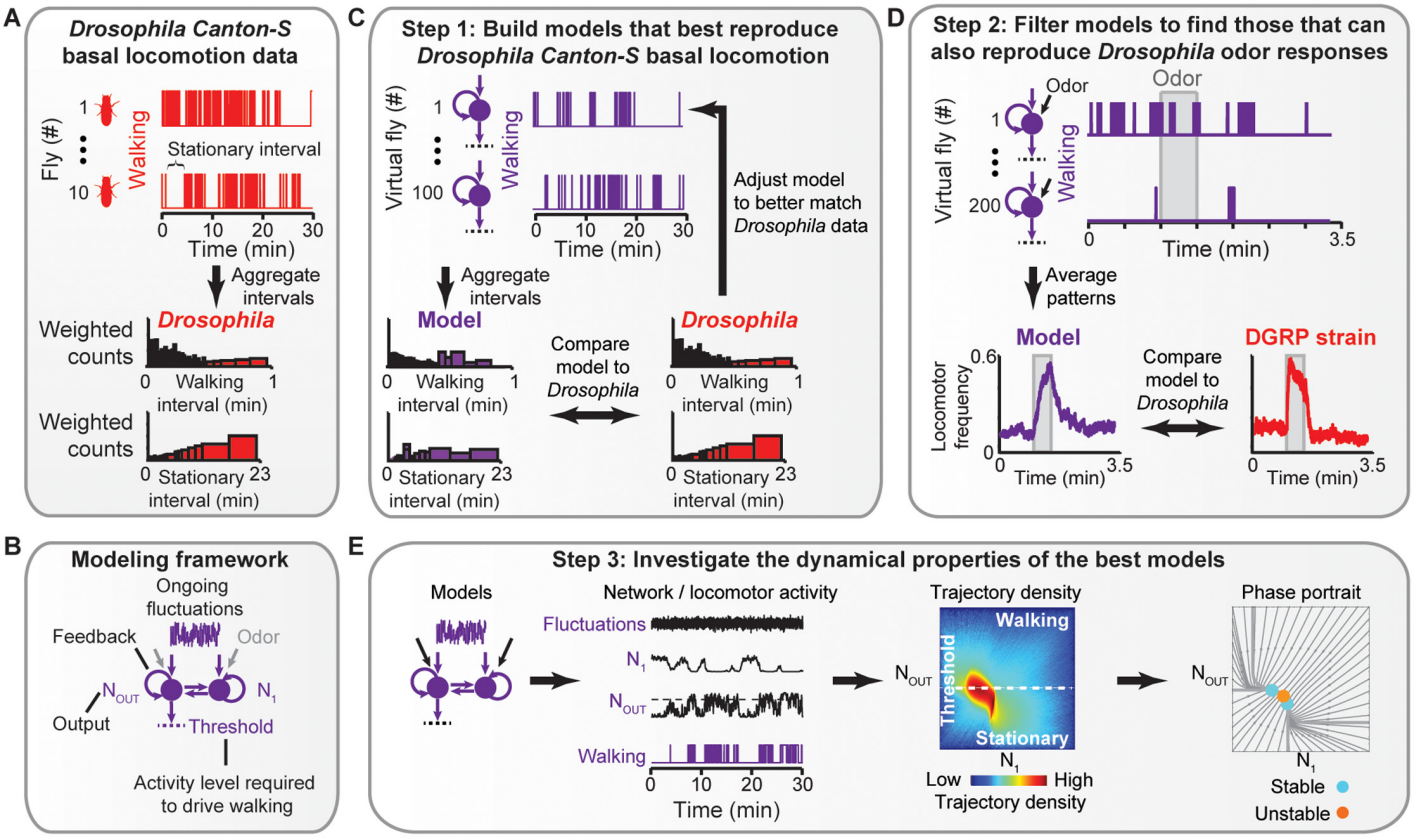
Workflow for generating and analyzing neural network models. (**A**) Basal locomotor patterns (filtered as in Fig 1D) are shown for two of ten *Canton-S* flies recorded for 30 min each. A stationary interval is highlighted for fly number ten. From these data, walking and stationary interval durations are aggregated and represented using weighted variable-width histograms for walking (top) and stationary (bottom) intervals. (**B**) A Continuous-Time Recurrent Neural Network (CTRNN) modeling framework used to investigate AS network dynamics. Models included up to five neurons, Gaussian noise inputs representing ongoing activity fluctuations, reciprocal and recurrent connections, odor inputs, and an output threshold to define the locomotor state of the virtual fly. (**C**) In the first step, a stochastic optimization approach is used to generate models that best reproduce *Drosophila* basal locomotor statistics. Locomotor statistics generated by a given model for 100 trials (virtual flies) are aggregated and compared to *Canton-S* strain data. The parameters for this model are then adjusted to reduce the difference from *Canton-S* data. This process is performed iteratively. (**D**) In the second step, odor inputs are added to the best previously generated models. For each model the strength of these inputs and the output threshold are adjusted iteratively to reduce the Root-Mean-Square Error (RMSE) between the model’s odor impulse locomotor pattern averaged across 200 trials (virtual flies) and a *Drosophila* strain’s odor impulse locomotor pattern averaged across ~200 flies. (**E**) In the third step, models that could match both *Canton-S* basal walking statistics and DGRP strain odor impulse locomotor patterns are characterized by the number of times specific neural activity levels are observed (‘Trajectory density’) color-coded from very frequent (dark red) to very rare (dark blue), and the tendency of neural activity, in the absence of fluctuations, to move toward (‘Stable’, cyan) or away from (‘Unstable’, orange) equilibrium activity levels (‘Phase portrait’). In each Trajectory density plot and Phase portrait, the bottom-left corner represents the lowest neural activity values.

We next asked to what extent fluctuations in AS neural activity can explain these common and reproducible locomotor properties, and what the underlying neural dynamics might be. To address these questions, we built neural network models that were constrained by the requirement to reproduce *Drosophila* basal and odor-evoked walking patterns. We took a three-step approach for generating and studying AS network models (Fig 3). First, we generated a population of models (Fig 3B) whose virtual locomotor outputs matched the statistics of basal locomotion for a single *Canton-S Drosophila* strain (Fig 3A and 3C). Second, from these models we identified those that could also reproduce the time-course of odor-evoked locomotion for three genetically distinct *Drosophila* strains (Fig 3D). Finally, we examined how the emergent dynamics of these networks allow them to reproduce *Drosophila* behavior (Fig 3E).

For the first step, we reasoned that basal locomotion in a sensory-deprived environment would most closely reflect the unperturbed, ongoing activity of *Drosophila* AS circuits. Therefore, we used these behaviors as a target dataset for neural network generation. In our initial experiments, we observed that some flies could remain stationary for over 20 min. Therefore, to capture the complete range of behavioral intervals, we acquired 5 h of basal locomotor sequences from *Canton-S* strain flies (Fig 3A).

The exact time-courses of individual fly behaviors depend on many, often unknown, factors. Therefore, we aimed to generate network models that could reproduce the duration of walking and stationary intervals rather than exact walking trajectories. Although flies spent more time near the arena edges [47] (S2 Fig), walking and stationary interval durations were only very weakly correlated with arena location (S2B–S2E Fig): the distance correlation [50] between interval start/end locations and interval durations were ~0.18 and ~0.08 for walking and stationary intervals, respectively (S2B–S2D Fig). These values roughly correspond to a 20% and 10% correlation between walking and stationary bout durations and locations (S2E Fig). Thus, locomotor patterns in our sensory-deprived environment were largely uncoupled from the arena geometry.

Our model-discovery method was based on stochastic parameter optimization and therefore required well-defined quantitative metrics for comparing candidate network models with *Drosophila* behavior (i.e., a cost function that guides the search for models). Additionally, to efficiently generate models, small changes to model parameters must result in similarly small changes in these quantitative metrics (i.e., a smooth fitness landscape) [51]. Therefore, we normalized histograms of walking and stationary interval durations in two ways. First, each histogram bin was multiplied by its own duration to ensure that more frequent, short-duration locomotor bouts were not over-valued. Second, empty bins were removed from each histogram by using variable bin widths (S3 Fig; see Materials and Methods). The resulting histograms provided a quantitative measure reflecting *Drosophila* basal locomotor patterns (Fig 3A, bottom).

To generate network models we employed a well-established neural network modeling framework, the Continuous-Time Recurrent Neural Network (CTRNN) [20]. CTRNNs are an intermediate representation of neural circuits that do not model precise ionic conductances or action-potential generation but retain the dynamical characteristics of neural circuits. Therefore, the emergent dynamics of generated network models, rather than their precise connectivity, are the instructive features [52]. Our CTRNN models were fully connected with recurrent and reciprocal connections between neurons, an intrinsic tau defining the time-scale of activity, and a bias input that constitutively drives the activity of each neuron. Depending on the experiment, our models could also have a Gaussian noise input—representing ongoing fluctuations in neural activity arising from both deterministic network dynamics as well as stochastic neuronal noise—and could have inputs representing olfactory sensory drive (Fig 3B). For each model, one neuron was selected prior to parameter optimization as the output neuron (N_OUT_) driving locomotor behavior. If this output neuron’s activity exceeded a threshold, the virtual fly was walking. Otherwise, the virtual fly was stationary.

Using this modeling framework (Fig 3B), we developed an automated pipeline to generate models whose virtual locomotor walking and stationary bouts had the same durations as the *Canton-S* strain basal locomotor bouts (Fig 3A). We used an iterative optimization algorithm, Particle Swarm Optimization [53], to define all network parameters (e.g., edge weights, tau, bias inputs) for multiple models in parallel (Fig 3C). To assess a new model, the optimization algorithm simulated it (i.e., a virtual fly) 100 times. An output threshold was then applied to the activity of the model’s output (N_OUT_), resulting in a binary (walking or stationary) time-series. We then aggregated the walking and stationary interval durations from this time-series and compared these histograms to the target *Canton-S* basal locomotion histograms. The optimization process allowed us to discover model parameters that minimize the difference between virtual basal locomotor patterns and *Canton-S* basal locomotor patterns. This value, ‘Difference from *Drosophila* data’, is a non-linear distance metric. It is therefore most intuitively understood by comparing it to values obtained when the full *Drosophila* dataset is compared to subsets of the same data (S4A Fig).

We generated models 50 times for each network size (1–5 neurons) either in the absence or presence of neural activity fluctuations. This resulted in 500 candidate models. In the second step, we further filtered this population of models by identifying those that could also replicate the complex odor-evoked locomotor patterns of three genetically distinct DGRP strains (Fig 2A). To mimic olfactory stimulation, we added virtual odor inputs to each model (Fig 3D). In the final step, we analyzed the emergent dynamics of models that best matched both basal and odor-evoked locomotor patterns by (i) identifying the most common neural activity levels for each model using a ‘trajectory density’ representation and (ii) performing dynamical systems analysis of each model in the absence of activity fluctuations to identify equilibrium points in phase space: activity levels that the network tended to settle towards (Stable) or move away from (Unstable) (Fig 3E). This revealed how activity fluctuations and dynamical properties allow models to reproduce complex *Drosophila* locomotor patterns.

### Small, fluctuation-driven models reproduce *Drosophila* locomotor patterns

**Fig 4:**
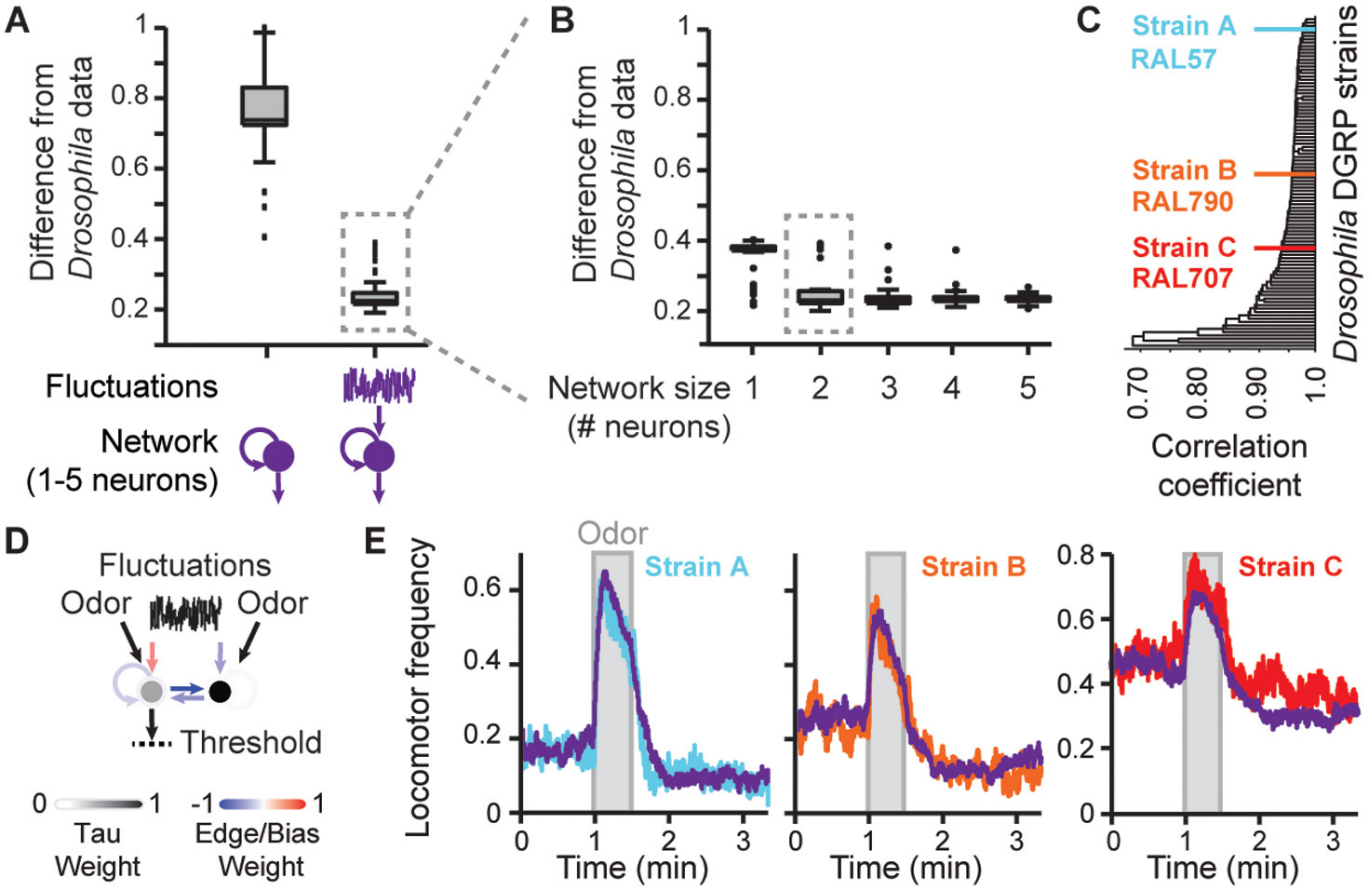
Small, fluctuation-driven models reproduce *Drosophila* locomotor patterns. (**A**) The capacity of discovered models without (left) or with (right) fluctuating inputs (n = 250 models for each condition with 50 models of each size) to reproduce the basal locomotor statistics of *Canton-S* flies. (**B**) Fluctuation-driven models from panel **A**, dashed box, separated as a function of network size (n = 50 models for each size). (**C**) A dendrogram illustrating the similarity of odor-evoked locomotor patterns across 98 DGRP strains. Hierarchical clustering distance was based on the Pearson’s correlation coefficient between odor-response time-series for each strain. The three strains chosen for further analysis are color-coded cyan (strain A—RAL57), orange (strain B—RAL790), and red (strain C—RAL707). (**D**) A graph representation of the best model overall in panel **B**. This model was chosen for all subsequent analysis. Recurrent and reciprocal connection strengths are color-coded. The tau value for each neuron is shown in grey-scale. (**E**) Odor impulse locomotor patterns for the model in panel **D** (purple) optimized to match the odor impulse locomotor patterns of DGRP strains A (RAL57), B (RAL790), and C (RAL707). Locomotor frequency time-series for each strain are color-coded cyan, orange, and red, respectively.

Using this approach we first asked if neural activity fluctuations were required to match the ongoing locomotor patterns of sensory-deprived *Drosophila*. Specifically, we tested if activity fluctuations were required by neural networks to reproduce *Canton-S* strain basal locomotor statistics (Fig 3C). Indeed, we found that fluctuations and network dynamics were both required by models to match these *in vivo* data (Fig 4A and 4B). Neither network dynamics alone (Fig 4A, n = 250 models with 1–5 neurons, *P* < 0.001, Wilcoxon Rank Sum Test; S4C–S4E Fig), nor a threshold applied to fluctuations in the absence of a network—the simplest, feed-forward AS model—performed as well (S4B Fig).

Fluctuation-driven networks with as few as two neurons accurately reproduced long and short time-scale *Drosophila* locomotor intervals (Fig 4B & S5A Fig). Notably, many of these two-neuron networks had similar dynamics. Each had two stable equilibrium points (S5B Fig) and could be further classified post-hoc based on the frequency with which neural activity visited each stable point (S5C Fig). Importantly, for all classes these equilibria did not represent a trivial mapping of two stable points onto two behavioral states (walking and stationary): both equilibrium points were below the threshold for walking (S5 Fig). Instead, in a manner akin to stochastic resonance in peripheral sensory pathways [4,6], walking bouts were engaged when activity fluctuations caused neural activity near the Up state to rise above the threshold for walking. These results reveal how surprisingly compact fluctuation-driven neural network models can reproduce complex *Drosophila* basal locomotor statistics spanning both long and short time-scales.

To identify the most explanatory of these network models, we tested their ability to match an unrelated behavioral dataset: the time-course of odor-evoked locomotor patterns across genetically distinct *Drosophila* strains. Of the original 98 DGRP strains, we selected three that spanned a large proportion of the behavioral variation that we observed (Fig 4C). Next, to keep their emergent dynamics unchanged, we left all network parameters fixed for the best performing network of each dynamical class (S5 Fig) while optimizing odor input strength and the locomotor output threshold to best match the time-course of odor-evoked locomotor patterns for each *Drosophila* strain (Fig 3D).

The best Class 1 model (Fig 4D) could faithfully reproduce the time-course of odor-evoked locomotor patterns for every *Drosophila* DRGP strain. It exhibited pre-odor basal locomotion, sharp increases in locomotor frequency at odor onset followed by a slow decay, and reduced post-odor basal locomotion (Fig 4E). Notably, not all models were as effective; the best Class 2 model failed to replicate odor-response dynamics (S6 Fig, Root-Mean-Square Error or RMSE > 0.08). The capacity for a given model to reproduce odor-evoked locomotor patterns was consistent across all three *Drosophila* strains (S6 Fig). In addition to fluctuations with Gaussian statistics—a standard modeling approach (e.g., [54])–our best Class 1 model could also match DGRP strain A locomotor patterns when driven by fluctuations with Power law, or Ornstein-Uhlenbeck (OU) statistics [54–56](S6C and S6D Fig).

### Dynamical mechanisms for reproducing *Drosophila* locomotor patterns

**Fig 5:**
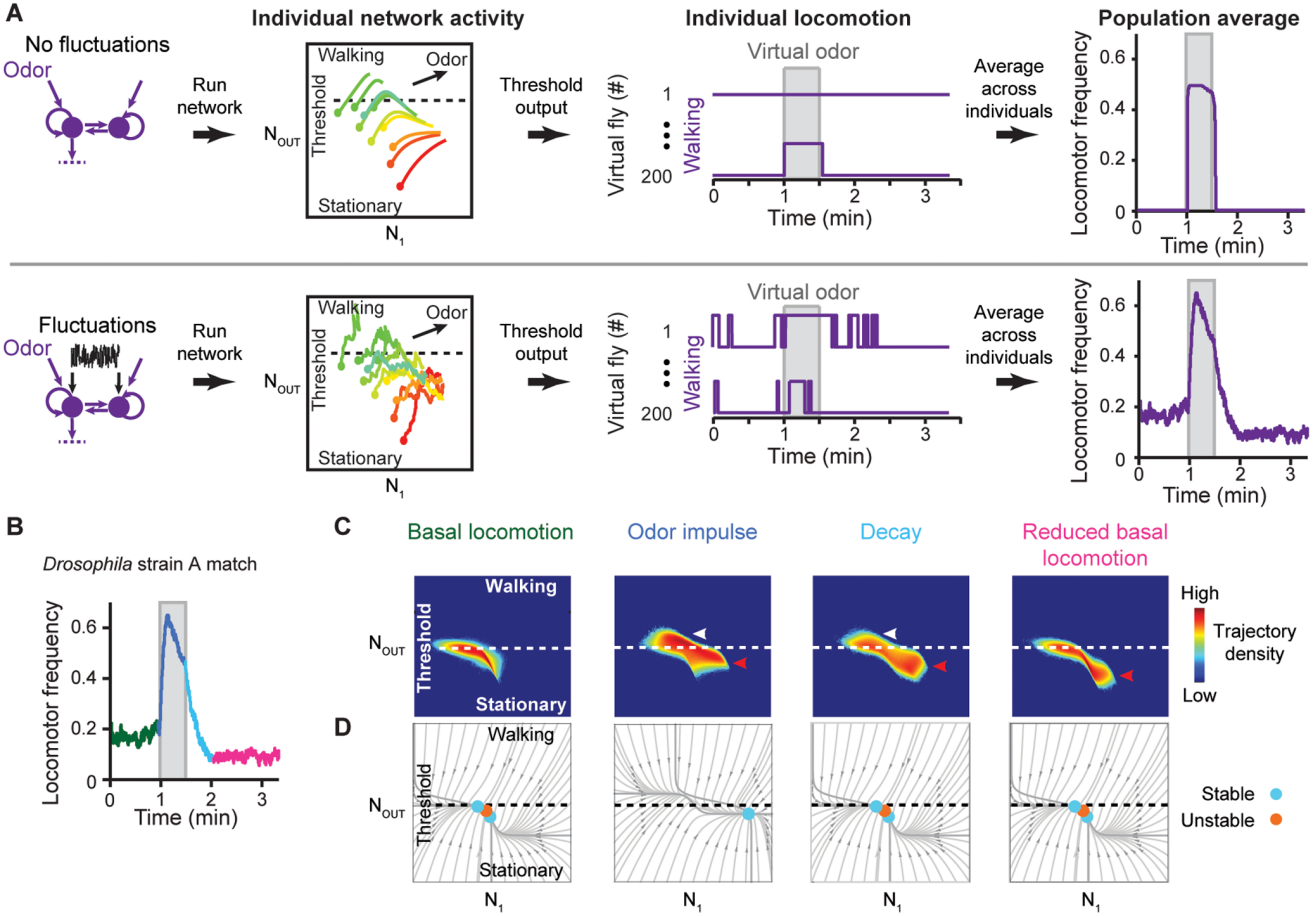
Dynamical mechanisms for reproducing *Drosophila* locomotor patterns. (**A**) Network activity, locomotion, and population-averaged locomotor patterns in the absence (top) or presence (bottom) of fluctuations. ‘Individual network activity’ plots show neural activity trajectories during odor stimulation starting from ten color-coded initial conditions. Solid circles indicate the starting points of each trajectory. ‘Individual locomotion’ plots show locomotor patterns for two representative virtual flies in the absence (top) or presence (bottom) of fluctuations. ‘Population average’ shows the locomotor frequency averaged across 200 virtual flies in the absence (top) or presence (bottom) of fluctuations. (**B**) Odor-evoked locomotor pattern of the best fluctuation-driven model (Class 1) tuned to match the locomotor pattern of *Drosophila* strain A. Color-coded are periods of pre-odor basal locomotion (green), odor impulse (blue), locomotor decay (cyan) and post-odor reduced basal locomotion (magenta). (**C**) Trajectory density plots and (**D**) phase portraits for this model during each time period indicated in panel **B**. In trajectory density plots, arrowheads highlight increased neural activity not observed during pre-odor basal locomotion. These are further labeled as being above (white) or below (red) the threshold for walking. In all phase portraits, grey lines with arrows are trajectories that indicate the direction of flow in phase space.

We next investigated how our best Class 1 model (S5D and S5E Fig) reproduced *Drosophila* basal and odor-evoked locomotor patterns (Fig 4E). We closely examined neural activity trajectories over time and identified several key roles for fluctuations. First, in the absence of fluctuations or sensory input, network activity remained trapped within stable equilibria below the threshold for walking (Fig 5A, top & S1 Video). By contrast, in the presence of fluctuations, neural trajectories could periodically and unpredictably transit between stable equilibria and sometimes exceed the activity threshold for walking (Fig 5A, bottom & S2 Video). Second, in our models, fluctuations were partially responsible for delayed changes in the dynamics of neural activity during and following odor removal. Rather than returning rapidly to stable equilibrium levels (Fig 5A, top ‘Individual network activity’), fluctuations caused neural activity to take a more tortuous path to these equilibria (Fig 5A, bottom ‘Individual network activity’). When averaged across a population of virtual flies, this results in a decay of locomotor frequency following odor stimulation (Fig 5A, compare top and bottom ‘Population average’).

Fluctuations by themselves were not sufficient, however, to explain the reduction in basal locomotion following odor stimulation. Traditionally, reductions in neural activity are often attributed to physiological depression due to over-stimulation [57]. However, we observed that several DGRP strains showed little (S7 Fig, RAL371) to no (S7 Fig, RAL642) odor-evoked increases in locomotion but still exhibited reductions in locomotor frequency following odor presentation. Since our models could reproduce post-odor reductions in locomotor frequency without physiological depression, we used these models to investigate how changes in neural dynamics might account for shifts in basal locomotion.

In our best Class 1 model matched to *Drosophila* strain A (RAL57), we discovered that odor stimulation caused a dramatic shift in network dynamics: the multistable network became monostable with a single subthreshold stable equilibrium point (Fig 5D, ‘Odor Impulse’). Therefore, although neural activity was initially pushed above the threshold by the odor (Fig 5C, ‘Odor impulse’, white arrowhead), its subsequent attraction to this new equilibrium point resulted in a decay of locomotor frequency even during odor stimulation (Fig 5C, ‘Odor impulse’, red arrowhead). When the odor was removed, although the network was once again multistable (Fig 5D, ‘Decay’), neural activity remained trapped near the odor-induced equilibrium point and took a long time to return to the original, basal equilibria (Fig 5C, ‘Reduced basal locomotion’, red arrowhead). This was due to both the diffusing influence of activity fluctuations as well as the structure of phase space. The same mechanisms allowed our best model to match *Drosophila* strain B (RAL790) (S8A–S8C Fig). Interestingly, even when two stable equilibrium points were retained, a substantial shift in the position of one stable point also resulted in decay dynamics matching those of *Drosophila* strain C (RAL707) (S8D–S8F Fig).

## Discussion

We have combined high-throughput behavioral analysis with automated neural network optimization to generate models that can reproduce complex *Drosophila* locomotor patterns. The resulting models, while not intended to inform the topology of *Drosophila* AS circuits, represent predictions about their emergent dynamics [19]. The key feature that allowed network models to reproduce *Drosophila* locomotor patterns was their dependence upon neural activity fluctuations. At first glance this may seem unsurprising given the complex nature of the behavioral data. However, fluctuations driving behavior in a simple feed-forward manner were insufficient (S4 Fig). Instead fluctuations required coupling to neural dynamics with two attributes.

**Fig 6:**
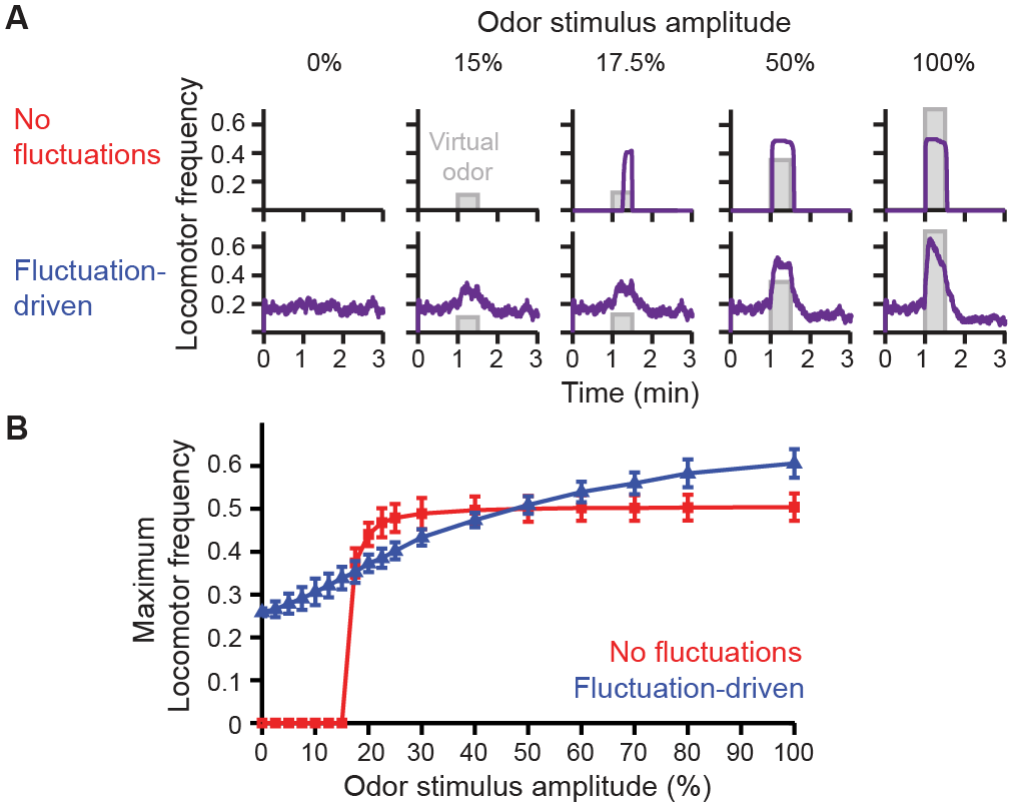
Fluctuations linearize the relationship between sensory drive and locomotor response. (**A**) Odor-evoked locomotor patterns as a function of odor stimulus amplitude for models without (top), or with ongoing fluctuations (bottom). (**B**) Maximum locomotor frequencies observed for models without (red), or with (blue) ongoing fluctuations at different odor stimulus amplitudes. Shown are the mean and standard deviations for 5 repetitions of each experiment.

First, our best models exhibited multistable dynamics reminiscent of persistent Up and Down states in vertebrate striatal [58] and cortical neurons [59]. Like stochastic state switching in genetic circuits [60,61] and stochastic mathematical models of *C*. *elegans* behavioral transitions [14], in our models fluctuations allowed neural activity to escape stable equilibria and to rise above the threshold for walking. Fluctuations near the Up state led to rapid bursts of walking while residence near the Down state led to longer periods of inactivity [28]. This is strikingly similar to stochastic resonance mechanisms observed in the sensory periphery [4,6]. There, noisy fluctuations uncover otherwise subthreshold sensory information. Similarly, in our networks, we found that fluctuations make it possible for weak sensory input to drive locomotion (Fig 6A). More generally, as for neurons in visual cortex [5], we observed that activity fluctuations linearize an otherwise nonlinear relationship between sensory drive and behavioral output (Fig 6B).

Second, in our best models, odor stimulation drove changes in network dynamics by shifting the position and, sometimes, number of stable equilibrium points. After the odor was removed the dynamics returned to the pre-odor state. However, neural activity trajectories were delayed in returning to these original basins of attraction due to both the structure of phase space as well as the diffusive influence of fluctuations. This represents an alternative dynamical mechanism for shifting circuit activity that complements well-studied molecular mechanisms like physiological depression [57]. Dynamical properties of a network can be pushed into a new regime through stimulation and, although the network is identical before and after stimulation, it produces a very different output. Therefore, the difference in pre- and post-odor *Drosophila* locomotor frequency may be explained by changes in the dynamic trajectories of a fixed system, without any modifications of synaptic strength.

One limitation of our study is the reliance upon one type of neural network model. CTRNN models are widely used and well-justified [19] but we expect that follow-up work using models with more [62] or less [63] detailed neural implementations can test the robustness of our predictions. In particular, highly-constrained models have been indispensible for understanding anatomically well-described systems like the pyloric network of the crustacean stomatogastric ganglion [64,65]. Although the anatomy and physiology of *Drosophila* AS circuits are not sufficiently well-characterized to build such detailed models, the body of anatomical and physiological data is growing [11,16,30,31]. This information will help to constrain neural network topologies [66–68] and to reveal how anatomical motifs contribute to the computation of *Drosophila* action timing.

Our dynamical models inform a long-standing debate about the relative influence of neural fluctuations on animal behavior [12,24]. Unlike in peripheral sensory circuits, fluctuations in central circuits may largely arise from deterministic signals that occur naturally within highly interconnected networks of neurons. Intriguingly, our models predict that fluctuating activity in central action selection circuits may act in a stochastic resonance-like manner [5] to linearize the relationship between sensory drive and behavioral output. This suggests a potentially beneficial role for neural fluctuations in increasing the dynamic range of sensory responses in complex environments.

## Materials and Methods

### *Drosophila* strains

*Drosophila Canton-S* strains were used in odor-impulse (Fig 1) and basal locomotion experiments (Fig 3A). *Drosophila melanogaster* Genetic Reference Panel (DGRP) [49] strains were used in odor-impulse experiments (Figs 2 & 4C and 4E).

### *Drosophila* behavior apparatus

Experimental arenas (50 mm x 10 mm enclosures with a height of 1.3 mm (Fig 1A–1C)) were designed using the 3D CAD software, SolidWorks (Dassault Systèmes, Waltham, Massachusetts, USA) and CNC machined from polyoxymethylene and acrylic glass. To backlight the arenas, we used a white LED panel (Lumitronix, LED-Technik GmbH, Hechingen) filtered with far-red semitransparent film (Eastman Kodak, Rochester, NY USA), a color for which fruit flies are visually insensitive [44]. For olfactory stimulation, we used air bubbled (Messer Schweiz AG, Lenzburg, Switzerland) through either water or 10% acetic acid and controlled using Mass Flow controllers (PKM SA, www.conab.gov.br) at a regulated flow rate of 500 mL/min via computer controlled solenoid valves (The Lee Company, Westbrook, CT, USA). We used a custom-fabricated circuit board and software [35] (sQuid, http://lis.epfl.squid/) to simultaneously control valves and acquisition cameras (Allied Vision Technologies, Stadtroda, Germany). We measured the flow of odor using a miniPID (Aurora Scientific Inc. Aurora, Ontario, Canada).

### *Drosophila* behavior experiments

We performed experiments on adult female *Drosophila* raised at 25°C on a 12 h light:12 h dark cycle at 2–5 days post-eclosion. Experiments occurred either the morning or late afternoon Zeitgeber Time. Prior to experiments, flies were starved for 4–6 h in humidified 25°C incubators. For odor stimulation experiments, we measured the locomotor behaviors of between 131 and 242 flies (median 205 flies). 98 DGRP strains were screened over the course of approximately 1 year. To minimize the effects of weekly and seasonal variation, we randomly selected and simultaneously screened groups of ~20 strains at a time. We repeated measurements for a single strain (RAL208) four times over the course of the screen to confirm season-independent behavioral reproducibility.

For basal locomotion behavior experiments, we recorded ten *Canton-S* strain flies for 30 min each, 5 h in total in a temperature-controlled room at 25°C under low red light illumination without air flow. Prior to the odor impulse experiment, flies were allowed to acclimate to the arena for 1 min. Subsequently, flies were first exposed to air throughout the arena for 1 min, then 10% acetic acid for 30 s, and finally, air for 90 s. Following an additional resting period with air flow for 90 s, we began the odor aversion experiment during which 10% acetic acid was presented on one side of the arena for 30 s and air on the other. This pattern alternated for an additional three cycles (Fig 1C, ‘Odor aversion’).

### *Drosophila* behavioral analysis

We measured each fly’s position over time using Ctrax and Matlab (The Mathworks, Natick, Massachusetts, USA) Behavioral Microarray software scripts [37]. Afterwards we discretized the speed of a fly into a binary time-series using a hysteresis threshold. Based on previous studies [26,28,47,48] and confirmed by our own measurements, we considered a fly to have begun walking when its speed exceeded 1 mm/s. For walking flies, we considered locomotion to have terminated when the speed decreased below 0.5 mm/s (a conservative value chosen to reduce the effects of measurement noise). We could thus classify speed in a binary fashion: walking or stationary (Fig 1D). When averaged over a population of flies, we obtained a ‘Locomotor frequency’: the proportion of active flies at a given time point ranging from 0 when no flies are walking, to 1 when all flies are walking (Fig 1F).

To calculate the reproducibility of basal locomotor frequencies for genetically identical groups of flies, we randomly sampled a group of 65 flies (selected as 50% of the flies from the strain with the smallest number of flies) from the same strain. We repeated this sampling 100 times per strain to measure the mean and standard deviation (Fig 2D).

To calculate the correlation between odor-response time-courses for fly strains, we randomly sampled two populations (groups A and B) of 65 flies (0.5* the minimum population size) from each strain. We then normalized Odor impulse traces (58^th^– 200^th^ s of the odor impulse experiment) between 0 and 1. Comparisons were performed either between groups from the same strain or from different strains. Each comparison was performed 100 times and the mean R^2^ value was plotted (Fig 2E).

To calculate odor aversion, for each fly we measured the proportion of time spent in the air zone minus the time spent in the odor zone over the course of the odor aversion experiment. This was divided by the total time of the aversion experiment yielding a value between -1 (always in the odor) and 1 (never in the odor) (Fig 2F and 2G).

To assess the effects of chamber geometry on the durations of *Drosophila* walking and stationary bouts, we computed the distance correlation (DC) [50] between either (a) walking or (b) stationary intervals start/end positions and their corresponding interval durations (S2B–S2D Fig). To increase the power of our analyses, we aggregated data-points by their positions with respect to one arena quadrant of the arena. To do this, data-points were folded twice—once along the Y-axis and a second time along the X-axis—to virtually aggregate them within one quarter of the arena. Consequently, all points near the four arena corners were considered near one another regardless of their corner of origin. For the sake of clarity, this repositioning is not shown in S2B–S2D Fig. To provide a reference metric for data with no correlation, we shuffled one of the variables and recomputed the DC. We repeated this process 100 times for each correlation. Additionally, to gain an intuitive understanding of DC values we took this shuffled dataset and introduced known correlations to incrementally larger subsets.

### Dendrogram generation

We generated a dendrogram representation of the correlation between odor impulse time-series across all 98 DGRP strains (Fig 4C) using an agglomerative hierarchical clustering algorithm. The algorithm performed single-linkage clustering using a distance function of 1 minus the sample correlation between points. The length of each branch represents the correlation between the odor impulse time-series of two strains of flies. For subsequent model matching we selected at random one strain from each of the following correlation intervals: ρ≤ 0.9, 0.9≤ρ≤0.95, ρ>0.95 (Fig 4C). We focused on only three DGRP strains due to the prohibitive computational time and resources required to optimize populations of virtual flies for each strain.

### Neural network modeling framework

For modeling we used Continuous-Time Recurrent Neural Networks (CTRNNs). This modeling framework was chosen for its ability to mimic the dynamics of biological neural circuits [17]. A CTRNN with *M* neurons *N*_1_,*N*_2_,…,*N*_*M*_ is defined by a system of ordinary differential equations (ODE):

$$\frac{dx_{i}}{dt}=F_{i}\left(t,\;x_{1},\;x_{2},\ldots ,\;x_{N}\right)=\;\frac{1}{\tau_{i}}\left(-x_{i}+\;\overset{M}{\underset{j=1}{\sum }}w_{ji}\cdot \sigma \left(x_{j}+b_{j}\right)+I_{i}\right),\;i=\left\{1,\ldots ,M\right\}$$

The state of a neuron *N*_*i*_ is defined by the variable *x*_*i*_ and is updated by an increment *dx*_*i*_ inversely proportional to the time constant *τ*_*i*_ ∈[0.05,50]. The output of a neuron *N*_*i*_ is obtained by evaluating a sigmoid transfer function $\sigma \left(x\right)=\;\frac{1}{\left(1+e^{-x}\right)}$ on the state *x*_*i*_ added to a constant bias *b*_*i*_ ∈ [−10,10]. Neurons *N*_*j*_ and *N*_*i*_ are connected with synaptic links of weight *w*_*ij*_ ∈ [−20,20]. Furthermore, each neuron can receive an optional input *I*_*i*_ (e.g., odor input). We tested models up to five neurons in size since even three neurons are sufficient to exhibit a wide variety of dynamical behaviors including chaos [69].

To investigate the capacity of activity fluctuations alone to match *Drosophila* basal locomotion, we optimized a threshold ranging from -4σ to 4σ directly upon a Gaussian noise source [2,70].

For network models without fluctuations (S4A Fig), the fluctuation input is set to zero (*I*_*i*_ = 0). For fluctuation-driven models, each neuron receives Gaussian noise with standard deviation *w*_*NOISE*_,_*i*_(Ii = *w*_*NOISE*_,_*I*_ *G*, where G ~ N(0, 1) follows a Normal distribution). To test the effects of different noise sources, we substituted Gaussian noise with either 1/f^α^ Power law noise (CNOISE, https://people.sc.fsu.edu/~jburkardt/m_src/cnoise/cnoise.html), or Ornstein-Uhlenbeck (OU) noise. OU noise was implemented following the standard formulation of an OU process:

$$dx_{t}=\theta \left(\mu -x_{t}\right)dt+\sigma \text{d}W_{t}$$

$$x\left(0\right)=x_{0}$$

where *W*_*t*_ represents the Wiener process. Using the best Class 1 model, we performed the odor impulse experiment while also optimizing the Power law parameter (α), or OU parameters (σ and θ).

We simulated CTRNNs using a custom high-performance C++ implementation. Our implementation used an approximation of the sigmoid function *σ*(*x*) [71] to speed-up simulations. Furthermore, to decrease the computational load of the simulations the noise value *G* was generated every *T*_*NOISE*_ ∈[0.01,1] s. For intermediate time-steps the noise value *G* was interpolated. Although this introduced correlations in the noise, the time-scale at which the noise value changed was orders of magnitude smaller than the time-scale at which the slowest dynamics occurred (hundreds of seconds). ODEs regulating the evolution of the CTRNN were integrated using ODEINT [72], a publicly available solver for ODE and a Runge-Kutta 4^th^-order method at a constant integration time-step of 10 ms (five times smaller than the smallest time constant of a neuron).

During the simulation of a neural network model, the trajectory of a model’s activity evolves over time from an initial condition, represented by the neuron states *x*_*i*_(*t*_*0*_), to eventually reside within the dynamical regime of the model (e.g., an equilibrium point, a limit cycle, etc.). In our experiments, we took two precautions to discard the long transients that sometimes occurred as trajectories passed from their initial positions into the model’s dynamical regime. First, we found the equilibrium points of the model (i.e., $\frac{dx_{i}}{dt}=0$). Then we generated initial conditions in the neighborhood of identified equilibrium points by sampling from a multivariate Gaussian distribution having an identity covariance matrix centered at the equilibrium points. Second, at the beginning of each simulation we integrated the model for 5 min of real time (3∙10^4^ time-steps) to discard dynamics during transit from initial conditions.

To generate a binary time-series equivalent to *Drosophila* walking and stationary bouts, we applied a threshold *THR* ∈ (0,1) to the output of a neuron, arbitrarily chosen to be *N*_0_ (referred to in the text as *N*_*OUT*_). Whenever the output of this neuron was greater than the threshold (*σ*(*x*_1_+*b*_1_)≥ *THR*), the virtual fly was walking and otherwise it was stationary.

### Neural network model parameter optimization

We optimized neural network model parameters using a stochastic optimization method for tuning model parameters in an iterative manner [53]. First, we generated a population of models of a given size (e.g., three neurons). Next, we measured the activity of these models and transformed these into binary time-series comprising walking and stationary bouts using a threshold. Finally, walking and stationary bout durations were measured and aggregated into weighted variable bin-width histograms for walking or stationary intervals. Bin-widths were derived from the *Drosophila* target dataset. We compared these histograms to target histograms measured from *Canton-S* flies. After assessing this population of models, parameters were adjusted towards those of the best performing models in this and previous iterations. The stochastic nature of this process ensured that final models were not identical to one another. This process was repeated until model performance converged. We then studied the topological and dynamical properties of the best models found.

In more detail, the *N*_*P*_ = {*w*_*ij*_,*τ*_*i*_,*b*_*i*_,*w*_*NOISE*_,_*i*_,*THR*,*T*_*NOISE*_| *i*,*j* ∈ *1*,…,*M*} parameters of the CTRNN models were optimized using Particle Swarm Optimization (PSO) [73]. We used standard parameter settings *c*_1_ = 2, *c*_2_ = 2. The inertia parameter *ω* of the algorithm was modified during an optimization run, following an update rule $\omega \left(t\right)=\;0.9\;-\;0.7\;*\;\frac{t}{T}$ to favor global search at the beginning of the optimization process and local search towards the end, where *t* is the current iteration and *T* = 200 is the maximum number of iterations. PSO operated concurrently on a set of *M* = 50 solutions. Therefore, a total of 10^4^ solutions were evaluated during each optimization run. Each function evaluation required between 11 s and 30 s of computational time. We optimized CTRNN models on a cluster (http://hpc.epfl.ch), using two nodes with 48 cores AMD Opteron 6176 (Magny-Cours) 2.3 GHz and 192 GB of memory.

To optimize the odor input strength and output threshold of our best models to match *Drosophila* odor impulse locomotor dynamics, we measured the activity of the model’s output during 60 s of no stimulation (basal locomotion), 30 s of odor stimulation, and then 120 s of no stimulation. We repeated this experiment while iteratively optimizing the few free parameters (odor input strength per neuron, and output threshold) to minimize the Root-Mean-Square Error (RMSE) between the target *Drosophila* odor-response time-series (average of ~200 flies) and the model’s odor-response time-series (average of 200 virtual flies).

### Measuring cost function values or the ‘Difference from *Drosophila* data’

The cost function assigns a score to each model evaluating how well it captures *Drosophila* locomotor patterns by comparing histograms generated by the model with histograms generated from *Canton-S* strain data. The comparison of these histograms is a crucial aspect of cost function design.

While it is possible to use standard statistical tests such as distance measures between empirical cumulative distributions of data (e.g., the Kolmogorov-Smirnov test), these statistical tests can mislead the optimization process by assigning reduced importance to rare events. For distributions of time durations, it is evident that these approaches would fail, since rare events (e.g., long walking or stationary periods) would be effectively ignored when comparing distributions. Therefore, we generated “weighted” histograms in which each bin was weighted by the duration it represents. For example, 10 walking events of 1 s duration and 1 event of 10s duration, would classically be represented as two bins of different “height” (10 and 1 respectively). In our weighted histograms these two bins have the same height (1*s*∙10 = 10s∙1).

We also wanted to remove empty bins. To do this we generated variable bin-width histograms. The boundaries of each bin for walking and stationary interval histograms were determined using *Canton-S* basal locomotion data (S3 Fig). We used the same bin boundaries when evaluating each neural network model.

For each cost function evaluation, we simulated a model *K* = 100 times. The model was started from *K* different initial conditions and simulated for 60 min of real time. For each of the *K* simulations, we selected at random with equal probability either the first or second 30 min of simulation, to mitigate overfitting of model behavior to the same trajectory and to foster model unpredictability. Each simulation produced a binary time-series representing walking (1) or stationary (0) behavior in a virtual fly. Thus, we computed the histogram for walking and for stationary periods using the data from all the selected *K* chunks. The generated histograms *H*_*s*,*w*_ for walking bouts and *H*_*S,I*_ for stationary bouts were compared to their respective target *Drosophila* histograms *H*_*T,W*_ and *H*_*T,I*_ obtaining the distance between the histograms *d*_*w*_ and *d*_*I*_.

$$d_{W}=\;d\left(H_{T,W},H_{S,W}\right)$$

$$d_{I}=\;d\left(H_{T,I},H_{S,I}\right)$$

The distance measure between a target and simulated histogram is defined as:

$$d\left(H_{T},H_{S}\right)=\;\overset{B}{\underset{i=1}{\sum }}\;\left|R\cdot h_{S}\left(i\right)\;-h_{T}\left(i\right)\right|\cdot t_{B}\left(i\right)$$

*B* is the number of bins in the histograms, *h*_*s*_,(*i*) and *h*_*T*_,(*i*) returns the count for bin *i* in the synthetic *H*_*s*_ and target histogram *H*_*T*_ and *t*_*B*_(*i*) returns the interval duration represented by bin *i*, here corresponding to the lower boundary of the bin. The scale factor *R* reconciles data obtained from simulations to available *Drosophila* data. We tested *K* = 100 simulated initial conditions per cost function evaluation. Therefore, *R* = 0.1 since we used data from 10 *Canton-S* strain flies.

The cost function *f* maps a model *m* to a cost function value in [0,∞], *f*:*m →* [0,∞]. For the sake of simplicity, we presented a normalized cost function value F. F is obtained by normalizing the cost function using the value *F*_*Norm*_ that a virtual fly would have if it is always walking or always stationary such that $F\left(m\right)=\frac{f\left(m\right)}{F_{Norm}}=\;\frac{d_{W}+d_{I}}{F_{Norm}}$. A value of 0 corresponds to a perfect match, a value of 1 corresponds to the score of a virtual fly that is always walking or always stationary. Intermediate values ranging between 0 and 1 correspond to plausible distributions. Values higher than 1 generally result from models with periodic dynamics at very high frequencies.

To derive an intuitive scale for cost function values, we evaluated values resulting from comparing subsets of *Drosophila* data with the full *Drosophila* dataset (S4 Fig). We generated subsets of data by selecting at random the desired number of flies F and replicating the data from each selected fly 10/F times, rounded up to the closest integer. In cases with too much data (F is not a divisor of 10), we randomly removed walking or stationary bouts until we obtained a dataset with the same length as the full *Drosophila* basal locomotion dataset.

### Generating variable bin-width weighted histograms

We computed the boundaries of variable bin-width weighted histograms using a vector *v* containing walking or stationary interval durations from 5 h of *Canton-S* basal locomotion. This routine took as inputs the minimum resolution *r* of a bin (the minimum separation between boundaries) and the minimum count *c* of events to generate a bin. Next it generated histogram boundaries by recursively splitting the initial single-bin boundaries [0,max(*v*)] into smaller bins containing a minimum of *c* events and having minimum duration of *r* seconds (S3 Fig).

First, we applied this procedure to get bin boundaries for *Drosophila* walking and stationary bout duration histograms (*H*_*T*,*W*_,*H*_*T*,*I*_). Then we used these same bin boundaries to compute both histograms from network model simulations (*H*_*S*,*W*_,*H*_*S*,*I*_).

### Dynamical systems stability analysis

Rather than studying their topologies (e.g., connectivity weights), we classified models by their dynamics [74–76]. In this way the behavior of a model with *n* neurons can be understood by observing the time evolution of its trajectory through an *n*-dimensional neural activity phase space. By studying the unfolding of phase space trajectories, one can identify common behavioral motifs among network models with widely different parameters. Using this formalism, features in phase space (e.g., attractors, limit cycles, and deterministic chaos) provide a clear language with which to interpret and compare different models [74]. This dynamical systems perspective has been successful in classifying both artificial neural networks and biological neural populations [23].

To analyze the global dynamical behavior of our models and to classify closely related ones, we performed stability analysis on our models in the absence of Gaussian noise. First, the *m* equilibrium points $\bar{x_{1}},\bar{x_{2}},\;\ldots ,\;\bar{x_{m}}$ of the CTRNN were identified by numerically finding the roots of the system of differential equations $F\left(\bar{x}\right)=0$ using the multi-dimensional root finder provided by the Gnu Scientific Library (http://www.gnu.org/software/gsl/). The Jacobian matrix *J* of a CTRNN is defined as:

$$J\left(x\right)=\left(\begin{array}{ccc} \frac{\partial F_{1}\left(x\right)}{\partial x_{1}} & \cdots & \frac{\partial F_{1}\left(x\right)}{\partial x_{M}}\\ \vdots & \ddots & \vdots \\ \frac{\partial F_{M}\left(x\right)}{\partial x_{1}} & \cdots & \frac{\partial F_{M}\left(x\right)}{\partial x_{M}} \end{array}\right)$$

$$\frac{\partial F_{i}\left(x\right)}{\partial x_{j}}=\{\begin{array}{cc} \frac{w_{ji}}{\tau_{i}}\frac{e^{x_{j}+b_{j}}}{{\left(1+e^{x_{j}+b_{j}}\right)}^{2}} & if\;i\neq j\\ -\frac{1}{\tau_{i}}+\frac{w_{ji}}{\tau_{i}}\frac{e^{x_{j}+b_{j}}}{{\left(1+e^{x_{j}+b_{j}}\right)}^{2}} & if\;i=j \end{array}$$

We studied the stability of the CTRNN by linearizing the system in the neighborhood of each equilibrium point and computing the eigenvalues of the Jacobian matrix *J* of the CTRNN for each equilibrium point $\bar{x}$ by solving $\text{det}\left(J\left(\bar{x}\right)-\lambda Ι\right)=0$. For a classification of stability given the equilibrium points’ eigenvalues refer to [74].

### Trajectory density maps

We obtained neural activity trajectory density maps by discretizing a plane described by two neuron states (*x*_*i*_,*x*_*j*_) into a grid of 10^3^ × 10^3^ cells ranging over the state values [−50,50]. Then we counted how many times a trajectory (its projection onto(*x*_*i*_,*x*_*j*_)) entered each cell. Density maps were generated by initializing models from 10^4^ random initial conditions. The color of a cell in the trajectory density plot is related to the logarithm of the probability that a neural activity trajectory passes through that cell.

### Network model classification

To quantify differences in the dynamical behavior of two-neuron fluctuation-driven models and to classify them, we generated 1000 initial conditions around each stable equilibrium point and let trajectories evolve for 30 min. We then counted how many times a trajectory switched from one equilibrium point to the other. Trajectories of Class 1 models switched many times during each 30 min period while Class 2 models switched more rarely. Trajectories of Class 3 models did not switch equilibrium points.

### Lyapunov exponent computation

We computed Lyapunov exponents for models in the absence of fluctuations (i.e., Gaussian noise) by integrating the variational equations $\frac{d\delta }{dt}$ of the CTRNN together with the original system:

$$\frac{d\delta }{dt}=\left(\begin{array}{ccc} \frac{d\delta_{11}}{dt} & \cdots & \frac{d\delta_{1M}}{dt}\\ \vdots & \ddots & \vdots \\ \frac{d\delta_{M1}}{dt} & \cdots & \frac{d\delta_{MM}}{dt} \end{array}\right)=\;J\left(\begin{array}{ccc} \delta_{11} & \cdots & \delta_{1M}\\ \vdots & \ddots & \vdots \\ \delta_{M1} & \cdots & \delta_{MM} \end{array}\right)$$

Following a standard procedure [77], we integrated the original system together with the variational equations for *T*_*LYAP*_ = *1000* time-steps. Then, we orthonormalized the perturbations using the Gram-Schmidt algorithm and computed the full spectrum of *M* Lyapunov exponents *λ*_*1*_ ≥ *λ*_*2*_ …≥ *λ*_*M*_. The Kaplan-Yorke dimension [78] was then computed as $D_{KY}=\;k+\overset{k}{\underset{i=1}{\sum }}\frac{\lambda_{i}}{\left|\lambda_{k+1}\right|}$, where *k* is the largest integer such that $\overset{k}{\underset{i=1}{\sum }}\lambda_{i}\geq 0$.

## Supporting Information

S1 Fig Binary classification of walking and the time course of odor flow. (**A**) A representative speed time-series for one *Canton-S* fly, classified as walking (red) or stationary (blue). High (1 mm/s) and low (0.5 mm/s) speed values for a hysteresis threshold are indicated (black dashed lines). (**B**) Histograms of speed data points taken from walking (red) and stationary (blue) intervals for 5 h of data from ten *Canton-S* flies. (**C**) Photoionization detector measurements (arbitrary units [au]) of odor flow (10% acetic acid). A high grey line indicates odor flow and a low grey line indicates air flow. Each colored trace represents one trial (*n* = 10). Both odor onset (top) and odor removal (bottom) are shown.
(TIF)

S2 Fig Relationship between basal locomotor intervals and arena position. (**A**) Basal locomotor trajectories of ten *Canton-S* flies within the arena over 5 h. Each black circle represents the location of one fly at one time-point. (**B-D**) The relationship between (**B**) walking interval start positions and interval durations, (**C**) walking interval end positions and durations, and (**D**) stationary interval start/end positions and durations. Intervals are color-coded by duration (top). Distance correlation values are shown below for the original data (black dashed line) and shuffled data (red boxplot, *n* = 100 each). (**E**) Distance correlation values for datasets in which incrementally larger correlations were introduced into shuffled data (*n* = 100 each) ranging from 10% (median DC ~0.1) to 100% (median DC ~0.9) of the data.
(TIF)

S3 Fig Procedure for generating and comparing weighted, variable-width histograms. (**A**) The procedure for determining bin-width sizes for variable bin-width histograms of *Canton-S* strain walking and stationary interval durations. (**B**) The workflow for generating weighted, variable bin-width histograms for *Canton-S* data (left) to compare with model data (right). Histograms were compared using a Root-Mean-Square Error (RMSE) to determine the cost function value or ‘Difference from *Drosophila* data’.
(TIF)

S4 Fig Cost function values for subsets of *Drosophila* data, fluctuations alone, and models without fluctuations. (**A**) *Canton-S* basal locomotion data as matched by increasingly larger time-normalized subsets of the same dataset. ‘*Drosophila* data used’ indicates the percent of flies selected and time-normalized to allow comparison with the full 5 h dataset from ten flies. *N* = 1000 datasets per boxplot. (**B**) The ability of a threshold applied to a Gaussian noise source (μ = 0, σ = 1) representing ongoing fluctuations to reproduce *Canton-S* basal locomotion data. The fluctuation time-step (i.e., noise correlation) is color-coded. Each data point is the lowest/best cost function value for a given threshold and a given fluctuation/Gaussian noise source. (**C**) The ability of models without fluctuations to reproduce *Canton-S* basal locomotion data. *N* = 50 models for each size ranging from 1–5 neurons. A black arrow indicates the best model in the absence of fluctuations. (**D**) A graph representation of the best model from panel **C**. Recurrent and reciprocal connection strengths are color-coded. The tau value for each neuron is shown in grey-scale. (**E**) Neural output activity (N_OUT_) and locomotor patterns for the best model from panel **C**. This model exhibits chaotic behavior (Largest Lyapunov Exponent = 0.011).
(TIF)

S5 Fig Classification of fluctuation-driven, two-neuron, multistable models. (**A**) The cost function value for each model sorted by class. The best model for each class is indicated (black arrow and outline). (**B**) Phase portraits for the best model from each class in panel **A**. Stable (cyan) and unstable (orange) equilibrium points are indicated. Grey lines with arrows are trajectories that indicate the direction of flow in phase space. The threshold between walking and stationary behavior is indicated (black dashed line). (**C**) The number of equilibrium points that neural activity trajectories visited over the course of 30 simulated min for 36 two-neuron, multistable models. *N* = 1000 simulations per model. Class 1 models visited each stable equilibrium point with high frequency. Class 2 models visited each equilibrium point a few times. Class 3 models visited only one equilibrium point. (**D**) Neural activity trajectory density plots for the best models in each class from panel **A**. The threshold between walking and stationary behavior is indicated (white dashed line). (**E**) A graph representation of the best models for each class from panel **A**. Recurrent and reciprocal connection strengths are color-coded. The tau value for each neuron is shown in grey-scale.
(TIF)

S6 Fig Capacity of best models from each class to reproduce *Drosophila* locomotor patterns. (**A**) Odor impulse responses for the best Class 2 two-neuron model (purple) tuned to match the odor impulse responses of DGRP strains A (RAL57), B (RAL790), and C (RAL707). Locomotor frequency time-series for each strain are color-coded cyan, orange, and red, respectively. (**B**) Root-mean-square error (RMSE) between odor impulse responses for the best model of each class and odor impulse responses for strains A, B, and C (cyan, orange, and red boxplots, respectively). *N* = 5 comparisons each. (**C**) Odor-impulse responses for the best Class 1 two-neuron model (purple) tuned to match the odor-impulse response of DGRP strain A (RAL57, cyan) when driven by fluctuations with Gaussian, Power law, or Ornstein-Uhlenbeck statistics. (**D**) Root-mean-square error (RMSE) between odor impulse responses for the best Class 1 model driven by fluctuations with Gaussian, Power law, or Ornstein-Uhlenbeck statistics and odor impulse responses for strain A. *N* = 5 comparisons each.
(TIF)

S7 Fig *Drosophila* strains exhibit reduced post-odor basal locomotion without substantial odor-evoked increases in locomotion. Locomotor traces averaged across 225 flies for DGRP strains (**A**) RAL371 and (**B**) RAL642 during the odor impulse experiment.
(TIF)

S8 Fig Network dynamics of the best Class 1 model matched to *Drosophila* strains B & C. (**A,D**) Odor impulse response for the best Class 1 model matching *Drosophila* strains B (**A**) and C (**D**). Color-coded are pre-odor basal locomotion (green), odor impulse (blue), post-odor locomotor decay (cyan), and reduced basal locomotion (magenta) periods. (**B,E**) Trajectory densities (top) and (**C,F**) phase portraits (bottom) for this model during each period. In all trajectory density diagrams, arrowheads highlight neural activity levels observed with more frequency than during pre-odor basal locomotion. These are further labeled as activity above (white) or below (red) the threshold for walking. In all phase portraits, grey lines with arrows are trajectories that indicate the direction of flow in phase space. The threshold between walking and stationary behavior is indicated in trajectory density plots (white dashed lines) and phase portraits (black dashed lines).
(TIF)

S1 Table RAL (Raleigh) strain number and mean pre-odor basal locomotor frequency for each DGRP strain. (XLSX)

S1 Video Activity of the best fluctuation-driven model (Class 1) before, during, and after odor stimulation, in the absence of fluctuations. The left panel shows the neural activity of neurons N_1_ (x-axis) and the output neuron N_OUT_ (y-axis) for 11 out of 200 virtual flies using the best model (two-neuron fluctuation-driven, Class 1) in the absence of fluctuations. The output threshold position is indicated (grey line). Each simulated virtual fly is color-coded. The bottom-right panel shows whether each of these virtual flies is walking or stationary. The top-right panel shows the locomotor frequency across the entire population of 200 virtual flies (purple) overlaid on top of the locomotor frequency measured across 198 flies of *Drosophila* strain A (cyan). Black vertical lines indicate the start and end of odor presentation, respectively.
(MOV)

S2 Video Activity of the best fluctuation-driven model (Class 1) before, during, and after odor stimulation, in the presence of fluctuations. The left panel shows the neural activity of neurons N_1_ (x-axis) and the output neuron N_OUT_ (y-axis) for 11 out of 200 virtual flies using the best model (two-neuron fluctuation-driven, Class 1) in the presence of fluctuations. The output threshold position is indicated (grey line). Each simulated virtual fly is color-coded. The bottom-right panel shows whether each of these virtual flies is walking or stationary. The top-right panel shows the locomotor frequency across the entire population of 200 virtual flies (purple) overlaid on top of the locomotor frequency measured across 198 flies of *Drosophila* strain A (cyan). Black vertical lines indicate the start and end of odor presentation, respectively.
(MOV)
