# Supplementary figures and images for: Fluctuation-Driven Neural Dynamics Reproduce Drosophila Locomotor Patterns
Source: PLoS Comput Biol. 2015 Nov 23;11(11):e1004577. doi: 10.1371/journal.pcbi.1004577 (PMC4657918; doi:10.1371/journal.pcbi.1004577)

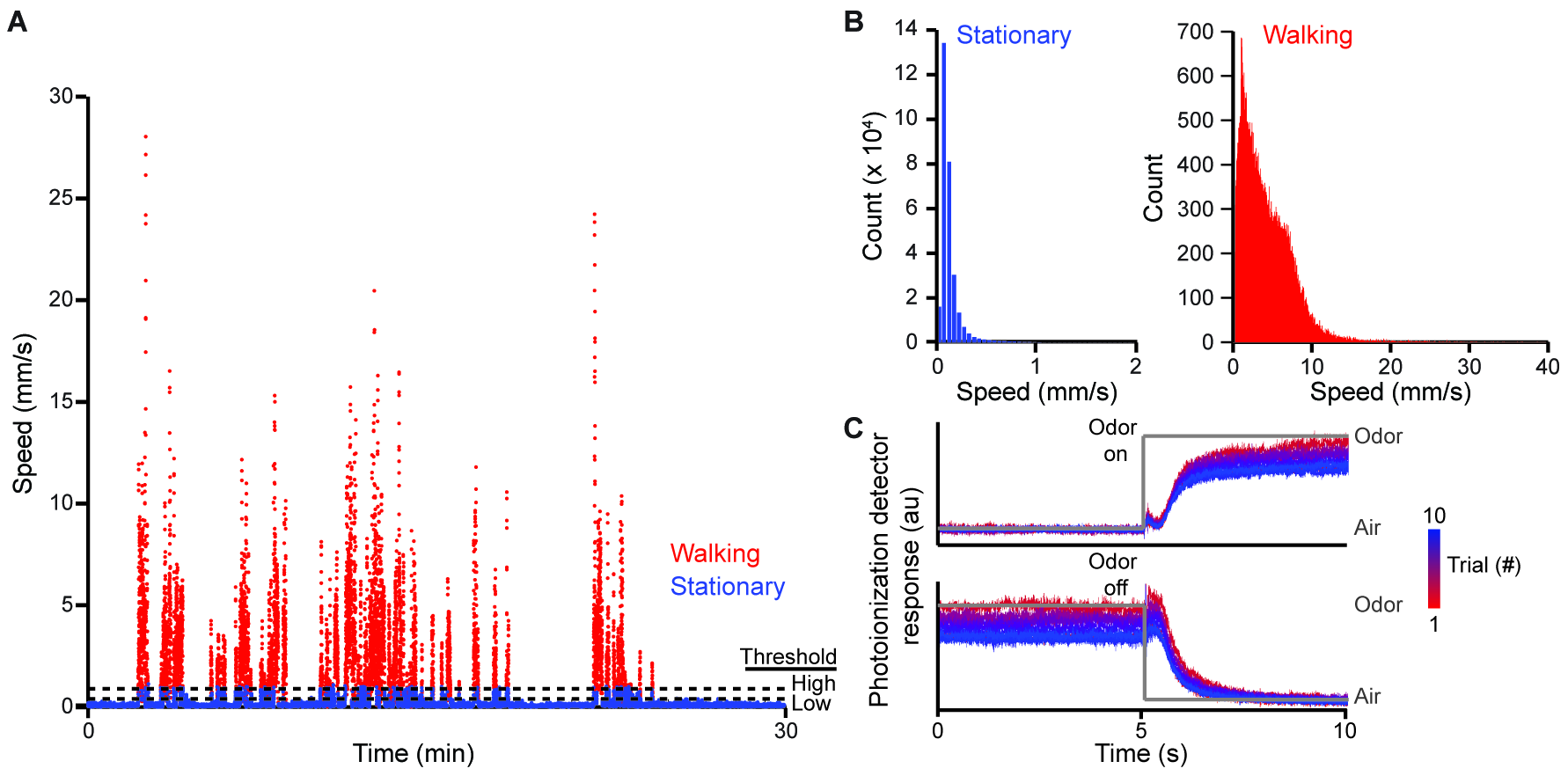

Supplement: S1 Fig — (A) A representative speed time-series for one Canton-S fly, classified as walking (red) or stationary (blue). High (1 mm/s) and low (0.5 mm/s) speed values for a hysteresis threshold are indicated (black dashed lines). (B) Histograms of speed data points taken from walking (red) and stationary (blue) intervals for 5 h of data from ten Canton-S flies. (C) Photoionization detector measurements (arbitrary units [au]) of odor flow (10% acetic acid). A high grey line indicates odor flow and a low grey line indicates air flow. Each colored trace represents one trial (n = 10). Both odor onset (top) and odor removal (bottom) are shown. (TIF) [file pcbi.1004577.s001.tif]

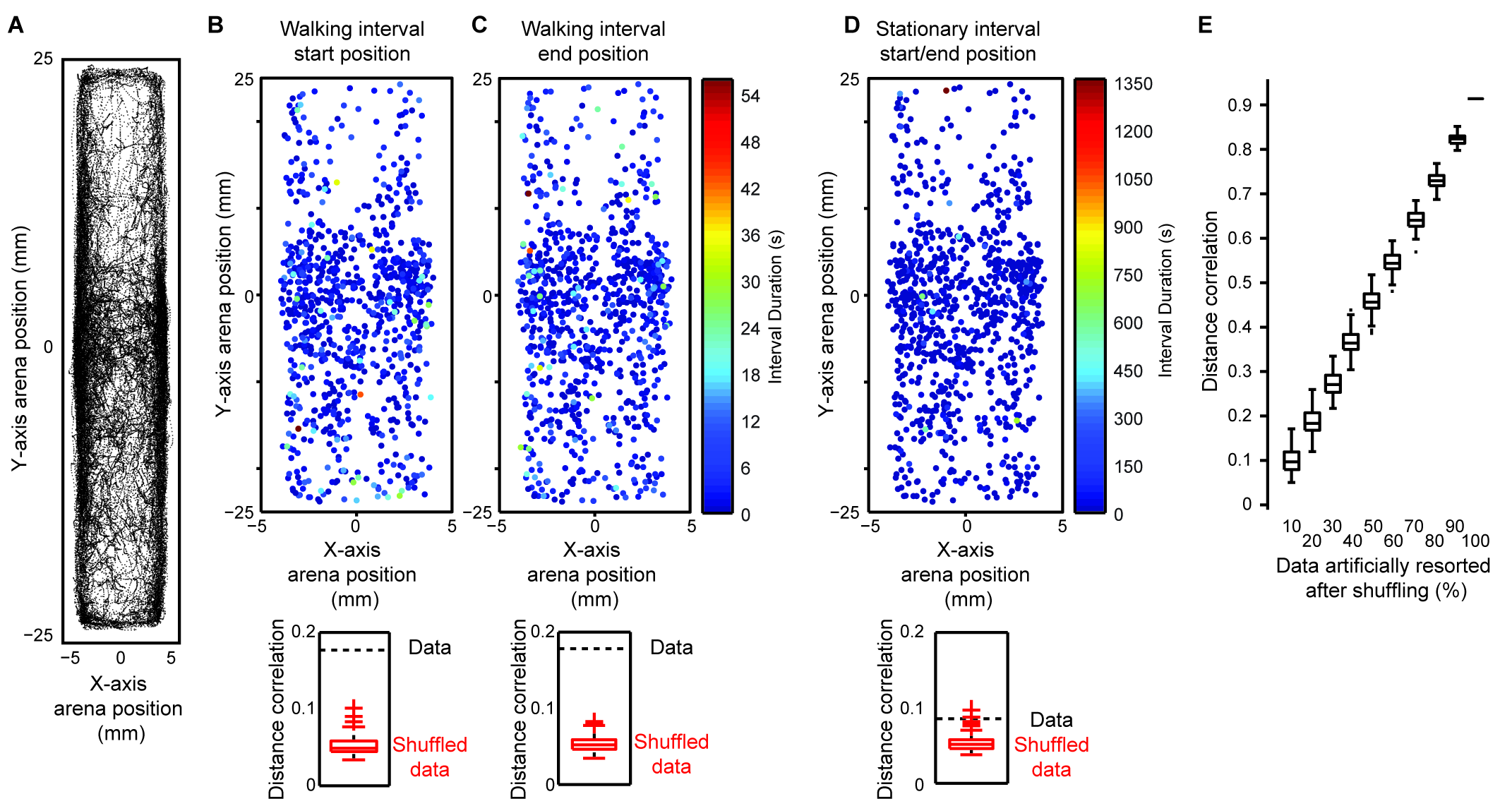

Supplement: S2 Fig — (A) Basal locomotor trajectories of ten Canton-S flies within the arena over 5 h. Each black circle represents the location of one fly at one time-point. (B-D) The relationship between (B) walking interval start positions and interval durations, (C) walking interval end positions and durations, and (D) stationary interval start/end positions and durations. Intervals are color-coded by duration (top). Distance correlation values are shown below for the original data (black dashed line) and shuffled data (red boxplot, n = 100 each). (E) Distance correlation values for datasets in which incrementally larger correlations were introduced into shuffled data (n = 100 each) ranging from 10% (median DC ~0.1) to 100% (median DC ~0.9) of the data. (TIF) [file pcbi.1004577.s002.tif]

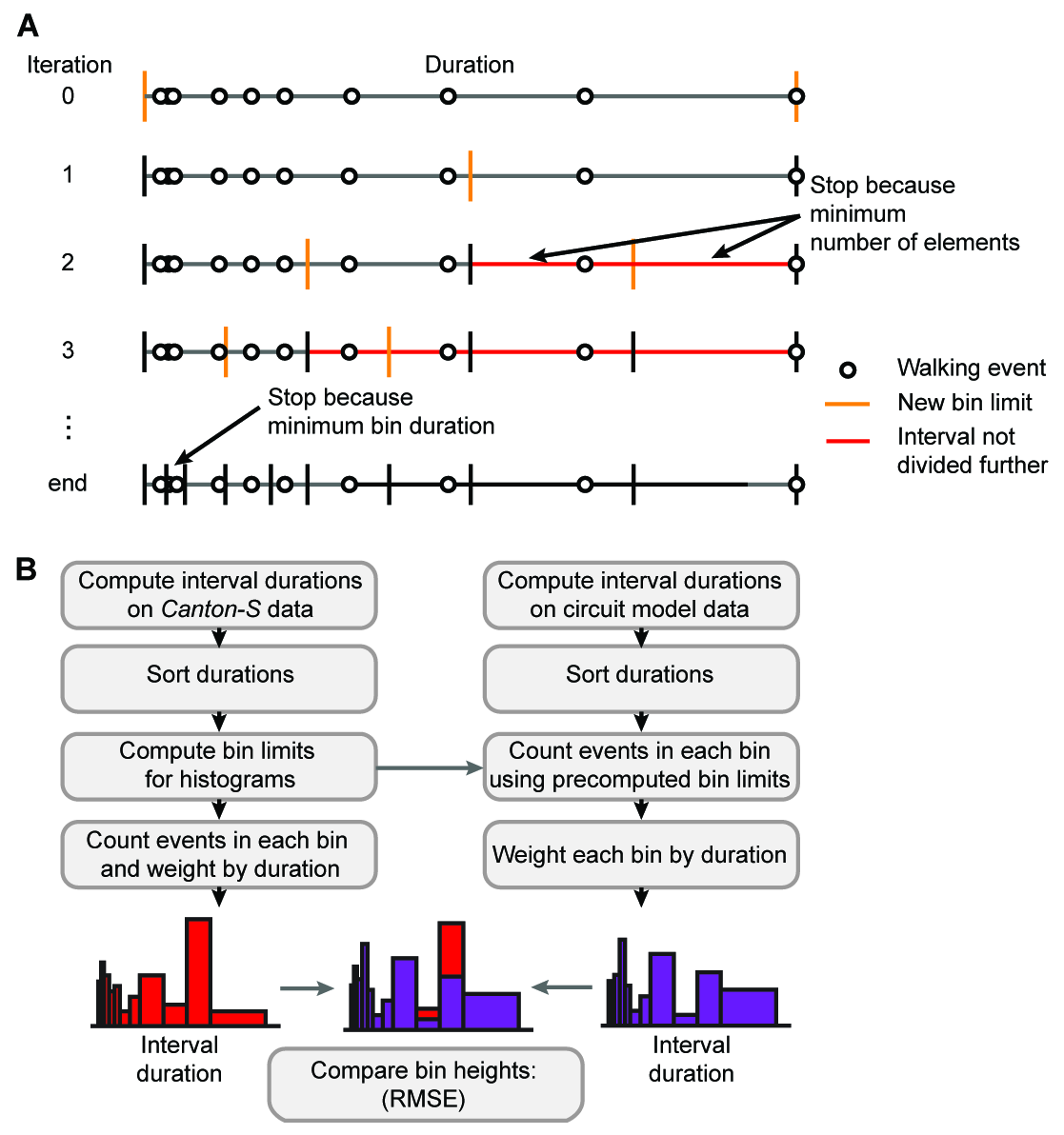

Supplement: S3 Fig — (A) The procedure for determining bin-width sizes for variable bin-width histograms of Canton-S strain walking and stationary interval durations. (B) The workflow for generating weighted, variable bin-width histograms for Canton-S data (left) to compare with model data (right). Histograms were compared using a Root-Mean-Square Error (RMSE) to determine the cost function value or ‘Difference from Drosophila data’. (TIF) [file pcbi.1004577.s003.tif]

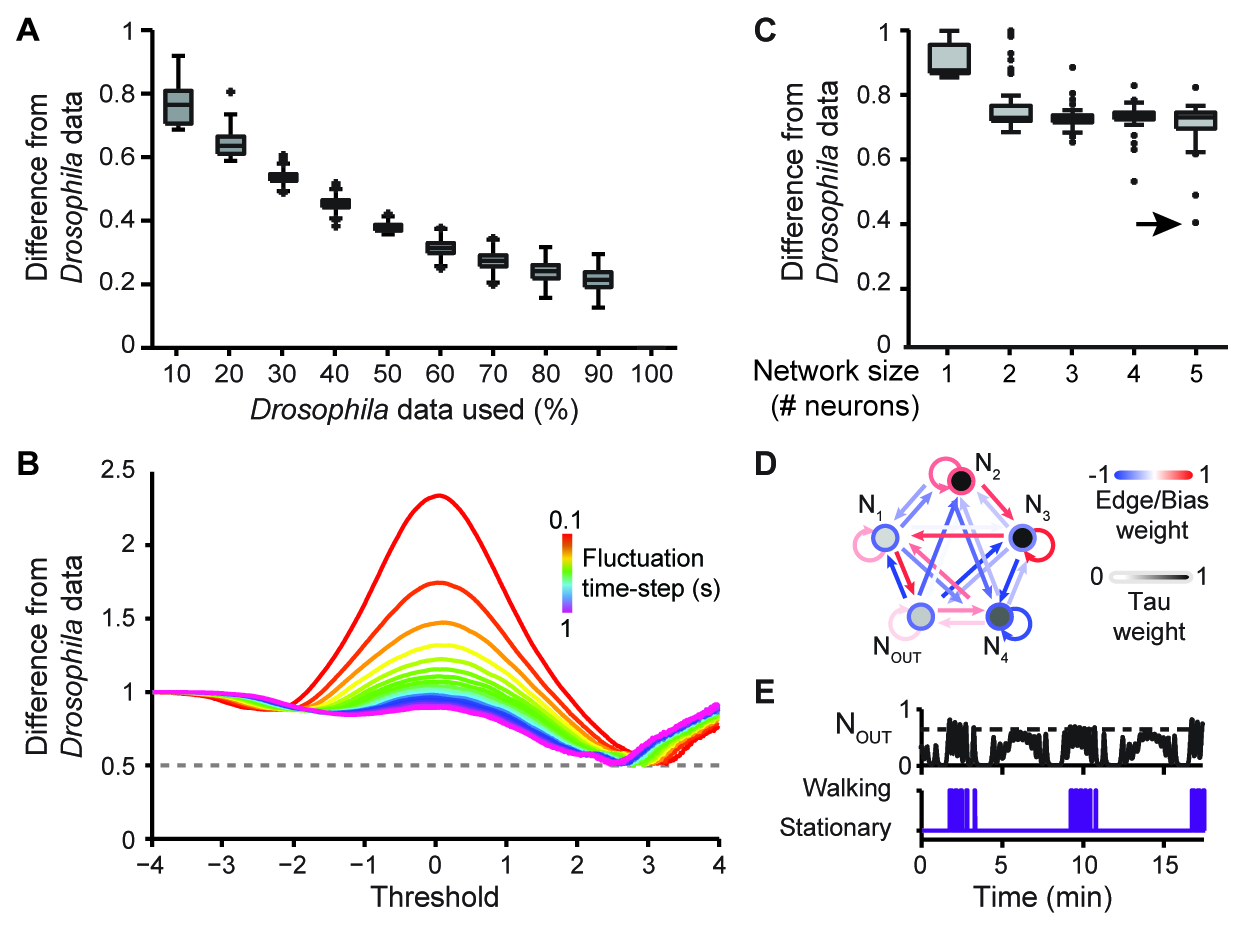

Supplement: S4 Fig — (A) Canton-S basal locomotion data as matched by increasingly larger time-normalized subsets of the same dataset. ‘Drosophila data used’ indicates the percent of flies selected and time-normalized to allow comparison with the full 5 h dataset from ten flies. N = 1000 datasets per boxplot. (B) The ability of a threshold applied to a Gaussian noise source (μ = 0, σ = 1) representing ongoing fluctuations to reproduce Canton-S basal locomotion data. The fluctuation time-step (i.e., noise correlation) is color-coded. Each data point is the lowest/best cost function value for a given threshold and a given fluctuation/Gaussian noise source. (C) The ability of models without fluctuations to reproduce Canton-S basal locomotion data. N = 50 models for each size ranging from 1–5 neurons. A black arrow indicates the best model in the absence of fluctuations. (D) A graph representation of the best model from panel C. Recurrent and reciprocal connection strengths are color-coded. The tau value for each neuron is shown in grey-scale. (E) Neural output activity (NOUT) and locomotor patterns for the best model from panel C. This model exhibits chaotic behavior (Largest Lyapunov Exponent = 0.011). (TIF) [file pcbi.1004577.s004.tif]

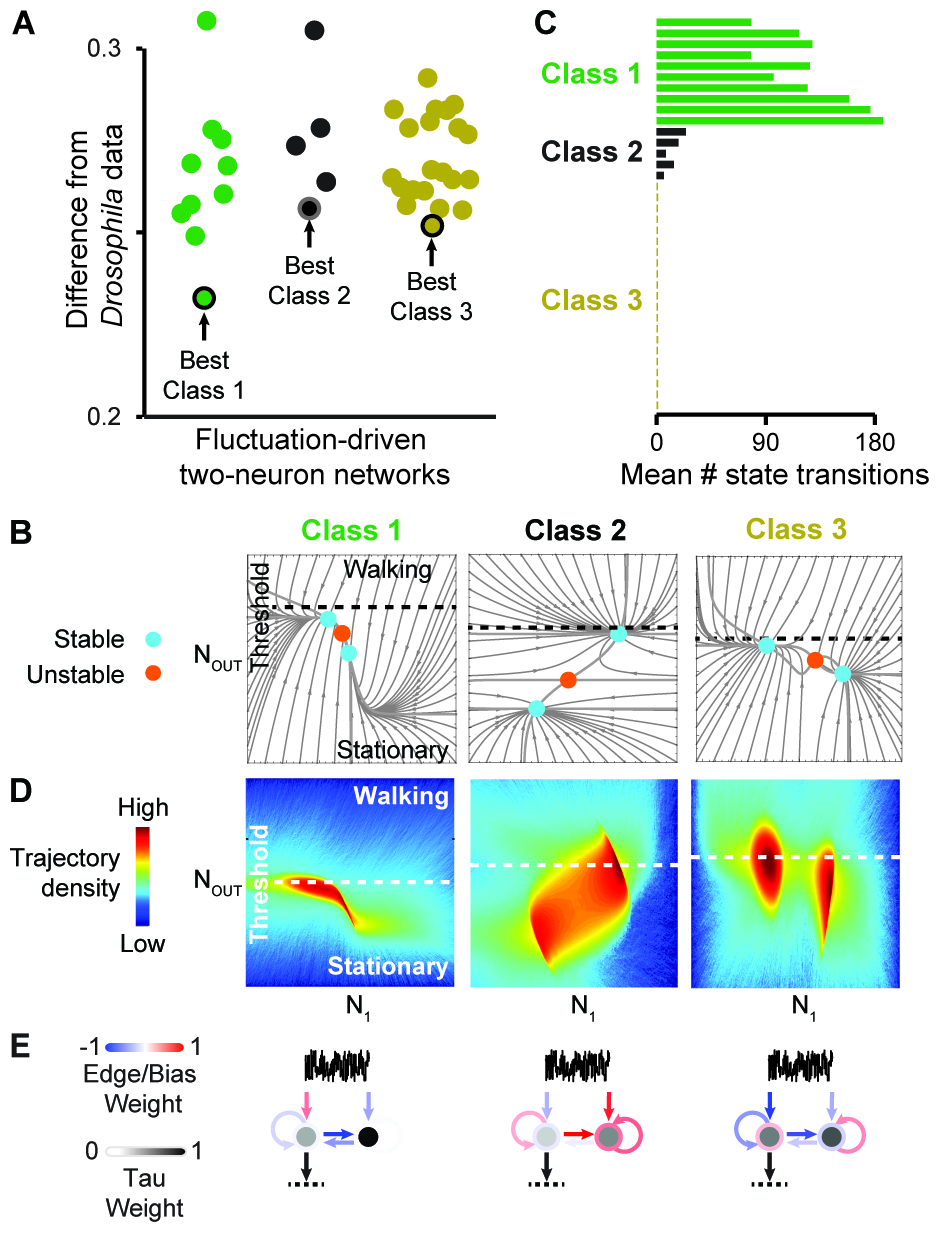

Supplement: S5 Fig — (A) The cost function value for each model sorted by class. The best model for each class is indicated (black arrow and outline). (B) Phase portraits for the best model from each class in panel A. Stable (cyan) and unstable (orange) equilibrium points are indicated. Grey lines with arrows are trajectories that indicate the direction of flow in phase space. The threshold between walking and stationary behavior is indicated (black dashed line). (C) The number of equilibrium points that neural activity trajectories visited over the course of 30 simulated min for 36 two-neuron, multistable models. N = 1000 simulations per model. Class 1 models visited each stable equilibrium point with high frequency. Class 2 models visited each equilibrium point a few times. Class 3 models visited only one equilibrium point. (D) Neural activity trajectory density plots for the best models in each class from panel A. The threshold between walking and stationary behavior is indicated (white dashed line). (E) A graph representation of the best models for each class from panel A. Recurrent and reciprocal connection strengths are color-coded. The tau value for each neuron is shown in grey-scale. (TIF) [file pcbi.1004577.s005.tif]

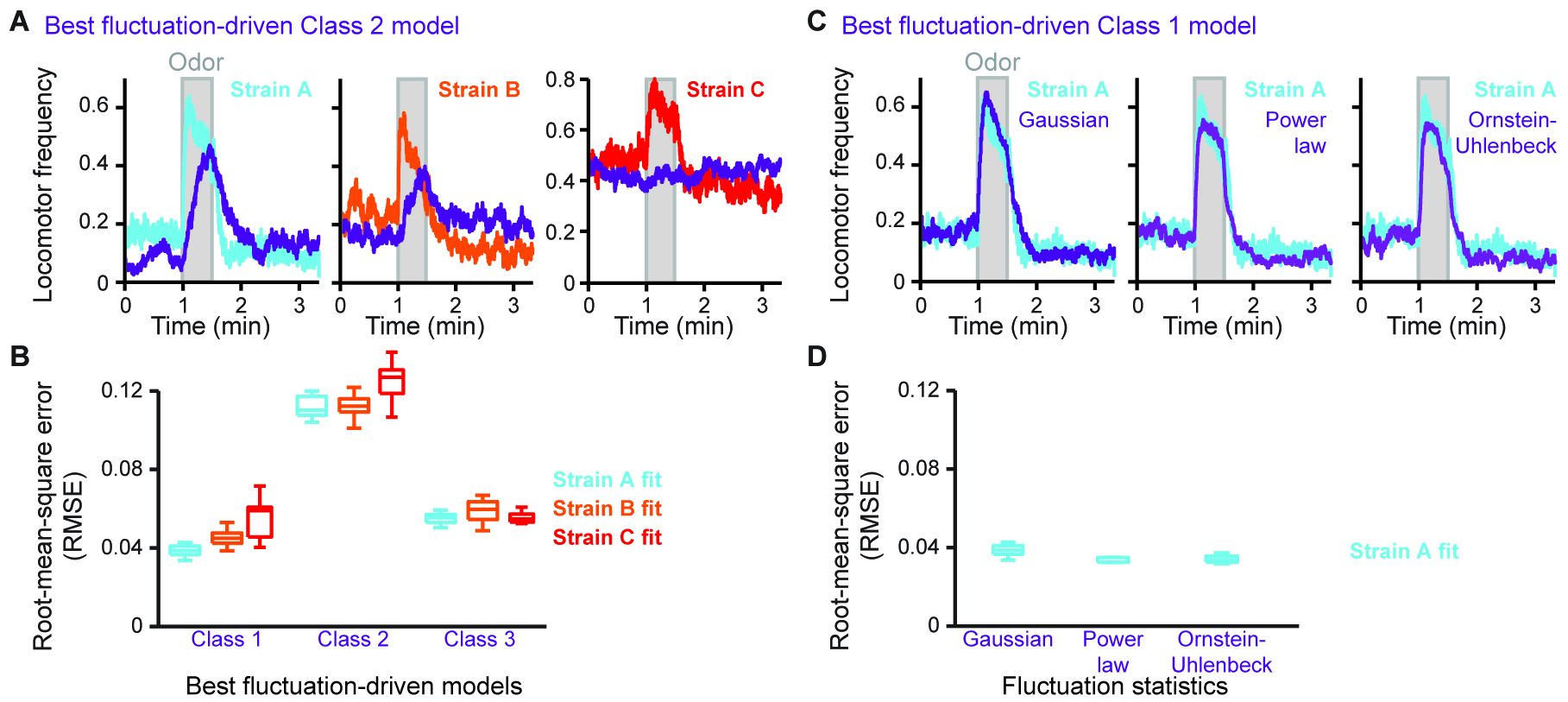

Supplement: S6 Fig — (A) Odor impulse responses for the best Class 2 two-neuron model (purple) tuned to match the odor impulse responses of DGRP strains A (RAL57), B (RAL790), and C (RAL707). Locomotor frequency time-series for each strain are color-coded cyan, orange, and red, respectively. (B) Root-mean-square error (RMSE) between odor impulse responses for the best model of each class and odor impulse responses for strains A, B, and C (cyan, orange, and red boxplots, respectively). N = 5 comparisons each. (C) Odor-impulse responses for the best Class 1 two-neuron model (purple) tuned to match the odor-impulse response of DGRP strain A (RAL57, cyan) when driven by fluctuations with Gaussian, Power law, or Ornstein-Uhlenbeck statistics. (D) Root-mean-square error (RMSE) between odor impulse responses for the best Class 1 model driven by fluctuations with Gaussian, Power law, or Ornstein-Uhlenbeck statistics and odor impulse responses for strain A. N = 5 comparisons each. (TIF) [file pcbi.1004577.s006.tif]

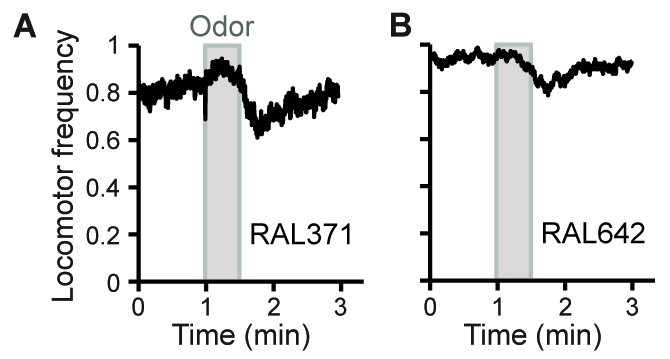

Supplement: S7 Fig — Locomotor traces averaged across 225 flies for DGRP strains (A) RAL371 and (B) RAL642 during the odor impulse experiment. (TIF) [file pcbi.1004577.s007.tif]

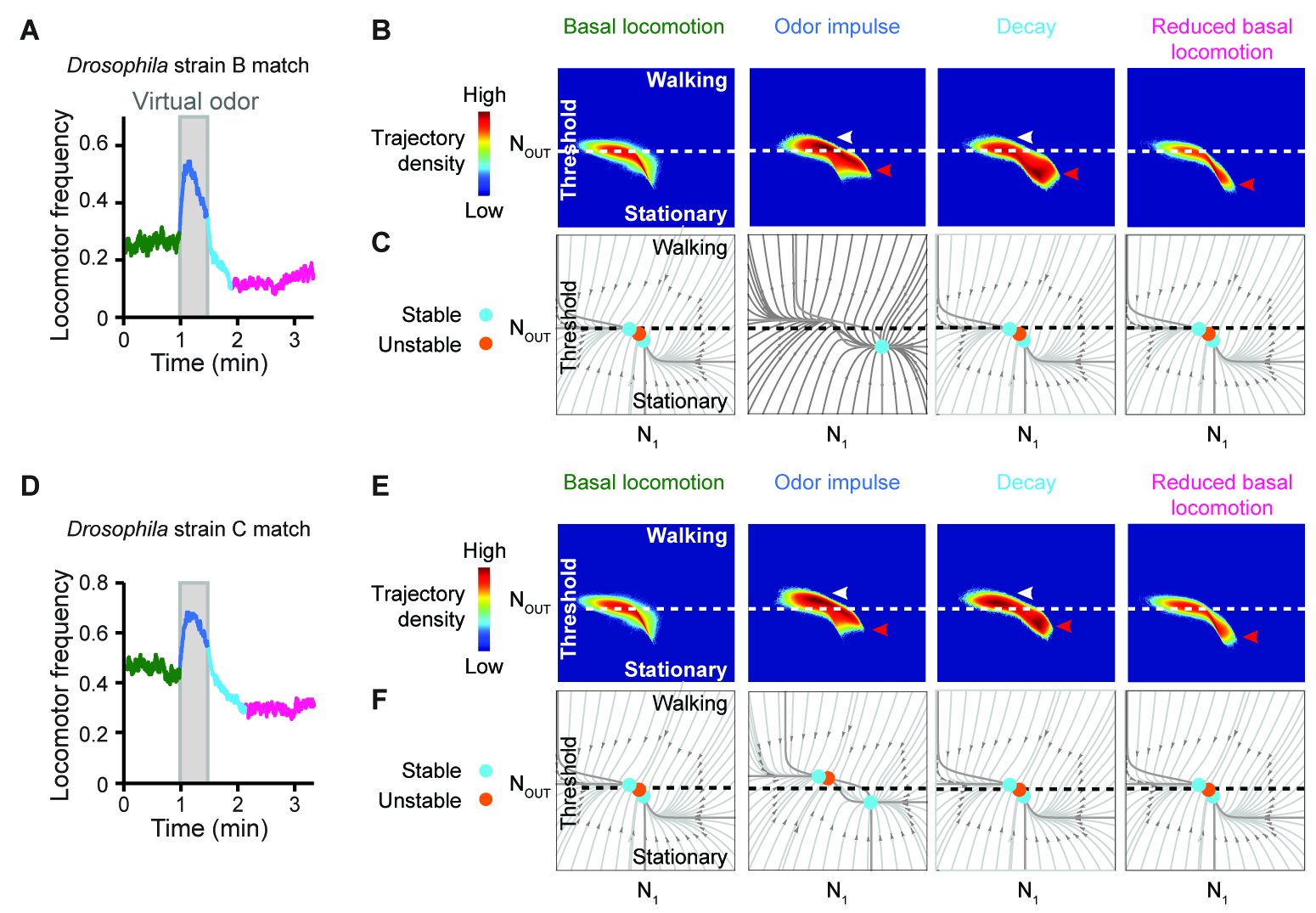

Supplement: S8 Fig — (A,D) Odor impulse response for the best Class 1 model matching Drosophila strains B (A) and C (D). Color-coded are pre-odor basal locomotion (green), odor impulse (blue), post-odor locomotor decay (cyan), and reduced basal locomotion (magenta) periods. (B,E) Trajectory densities (top) and (C,F) phase portraits (bottom) for this model during each period. In all trajectory density diagrams, arrowheads highlight neural activity levels observed with more frequency than during pre-odor basal locomotion. These are further labeled as activity above (white) or below (red) the threshold for walking. In all phase portraits, grey lines with arrows are trajectories that indicate the direction of flow in phase space. The threshold between walking and stationary behavior is indicated in trajectory density plots (white dashed lines) and phase portraits (black dashed lines). (TIF) [file pcbi.1004577.s008.tif]
